# Diverse Kir Expression Contributes to Distinct Bimodal Distribution of Resting Potentials and Vasotone Responses of Arterioles

**DOI:** 10.1371/journal.pone.0125266

**Published:** 2015-05-04

**Authors:** Yuqin Yang, Fangyi Chen, Takatoshi Karasawa, Ke-Tao Ma, Bing-Cai Guan, Xiao-Rui Shi, Hongzhe Li, Peter S. Steyger, Alfred L. Nuttall, Zhi-Gen Jiang

**Affiliations:** 1 Oregon Hearing Research Center, Oregon Health & Science University, Portland, OR, 97239, United States of America; 2 Department of Biology, South University of Science and Technology of China, Shenzhen, 518055, China; 3 Department of Physiology, Shihezi University Medical College, Shihezi, China; 4 Department of Pharmacology, Hebei Medical University, Shijiazhuang, China; VCU, UNITED STATES

## Abstract

The resting membrane potential (RP) of vascular smooth muscle cells (VSMCs) is a major determinant of cytosolic calcium concentration and vascular tone. The heterogeneity of RPs and its underlying mechanism among different vascular beds remain poorly understood. We compared the RPs and vasomotion properties between the guinea pig spiral modiolar artery (SMA), brain arterioles (BA) and mesenteric arteries (MA). We found: 1) RPs showed a robust bimodal distribution peaked at -76 and -40 mV evenly in the SMA, unevenly at -77 and -51 mV in the BA and ~-71 and -52 mV in the MA. Ba^2+^ 0.1 mM eliminated their high RP peaks ~-75 mV. 2) Cells with low RP (~-45 mV) hyperpolarized in response to 10 mM extracellular K^+^, while cells with a high RP depolarized, and cells with intermediate RP (~-58 mV) displayed an initial hyperpolarization followed by prolonged depolarization. Moderate high K^+^ typically induced dilation, constriction and a dilation followed by constriction in the SMA, MA and BA, respectively. 3) Boltzmann-fit analysis of the Ba^2+^-sensitive inward rectifier K^+^ (Kir) whole-cell current showed that the maximum Kir conductance density significantly differed among the vessels, and the half-activation voltage was significantly more negative in the MA. 4) Corresponding to the whole-cell data, computational modeling simulated the three RP distribution patterns and the dynamics of RP changes obtained experimentally, including the regenerative swift shifts between the two RP levels after reaching a threshold. 5) Molecular works revealed strong Kir2.1 and Kir2.2 transcripts and Kir2.1 immunolabeling in all 3 vessels, while Kir2.3 and Kir2.4 transcript levels varied. We conclude that a dense expression of functional Kir2.X channels underlies the more negative RPs in endothelial cells and a subset of VSMC in these arterioles, and the heterogeneous Kir function is primarily responsible for the distinct bimodal RPs among these arterioles. The fast Kir-based regenerative shifts between two RP states could form a critical mechanism for conduction/spread of vasomotion along the arteriole axis.

## Introduction

Auditory transduction is associated with heavy energy consumption [1], and is extremely vulnerable to vascular disturbances. Loud sounds decrease cochlear blood flow (CBF), yet moderate intensity sound exposure increases CBF [2,3]. Anoxia or interruption of CBF drastically reduces cochlear function [4,5]. Accumulated evidences suggest that vascular malfunction contributes to age-related hearing loss [6,7,8], Meniere’s disease [9], some forms of sudden deafness [10] and increased risk of drug- and noise-induced ototoxicity [11]. To effectively treat those hearing disorders, a better understanding of how CBF is regulated is critical.

**Fig 1:**
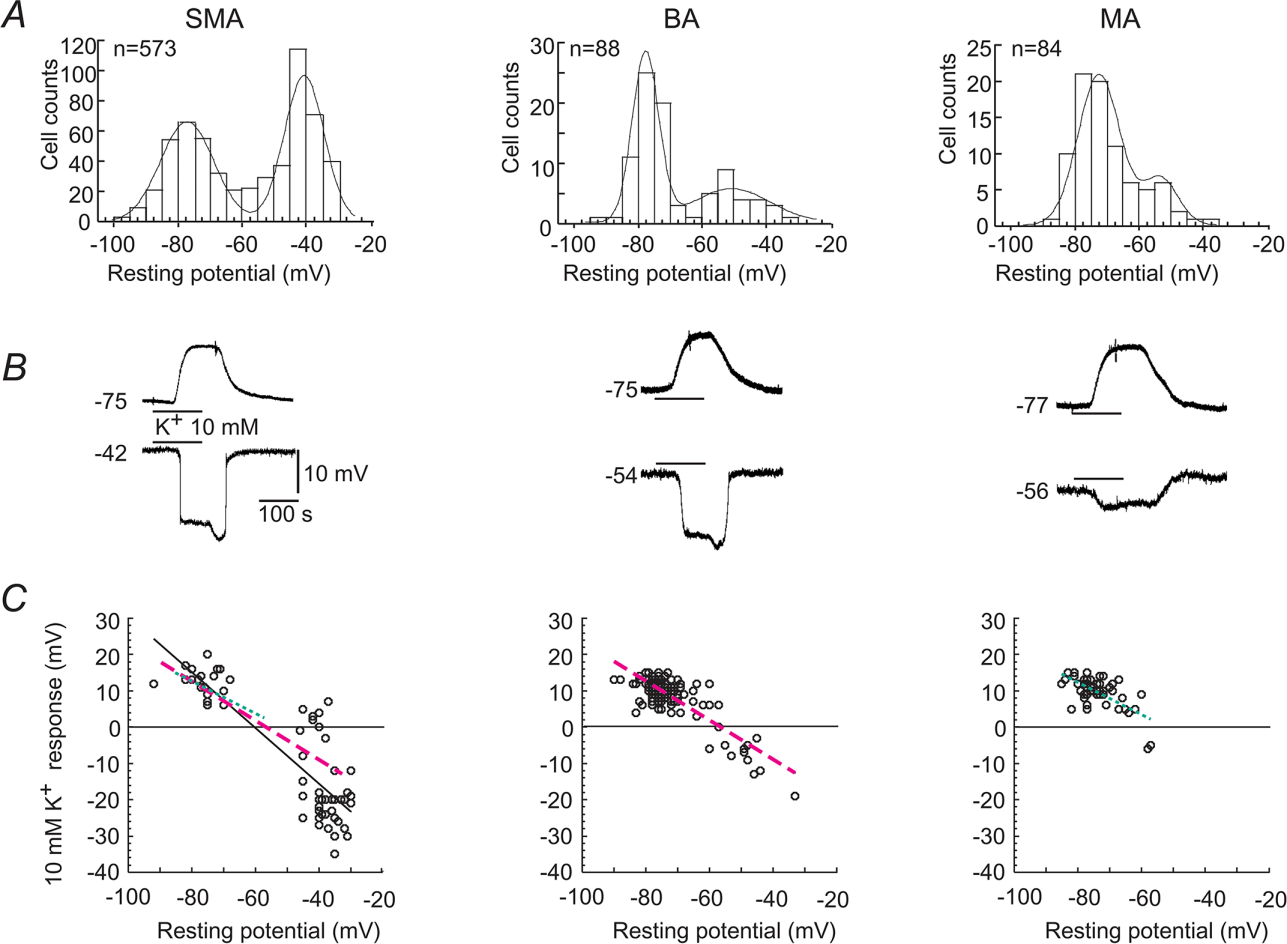
Distribution of RPs and responses to high K^+^ in the SMA, BA and MA arterioles. (***A***) The initial RPs of cells sampled from each vessel type displayed in 5 mV bin histograms. The frequency curves are modeled with a mixture of two Gaussian functions [16]. (***B***) Representative membrane potential responses to 10 mM K^**+**^ of high (top row) and low (bottom row) RP cells in each vessel type. The estimated E_K_ was -86 and -68 mV in 5 and 10 mM K^**+**^ bath solutions, respectively. (***C***) The plots of depolarization and hyperpolarization responses to 10 mM K^**+**^ against the initial RP for individual cells randomly sampled from each vessel type. Linear regression line to data is dash-coded for each vessel type: bold solid line for the SMA, thick and thin dash lines for BA and MA, respectively. The latter two are duplicated in the SMA panel for comparison.

The cochlear spiral modiolar artery (SMA) is of particular interest because it is the primary blood supplier to the cochlea [12]. The SMA of rodents is 30–80 μm in diameter, with a single layer of vascular smooth muscle cells (VSMCs), and classified as arterioles [13,14,15]. We have found several surprising properties of the cells in isolated segments of the SMA. In particular, we demonstrated that the resting potentials (RP) of these cells show a robust bimodal distribution, peaking at ~-40 and -75 mV, called low and high RP, respectively [16] (see also Fig 1*A*).

Intracellular recordings show that the RP may rapidly shift from a low to a high level, and vice versa; thus the bimodal peaks reflect bi-stable RP states. The shift between these two distinct RP levels is mimicked by wash-in and wash-out of micro-molar concentrations of barium (Ba^2+^) ions, and the shift often occurs with a regenerative (positive feedback) fast phase, indicating that the bi-stable status is generated by an all-or-none-like activation/deactivation of the inward rectifier K^+^-channel (K_ir_) [16].

It is known that the RP of VSMCs is a major determinant of cytosolic calcium concentration and thus the vascular tone [17]. In small arteries, vascular tone may be related to the degree of depolarization without involvement of action potentials in VSMCs [18,19]. In pressurized *in vitro* or *in vivo* small arteries, the cells normally have a RP approximately -40 mV [20,21,22], with no apparent bimodal distribution. However, a big swift RP shift in a conducted hyperpolarization and dilation has often been observed during an endothelial derived hyperpolarizing factor (EDHF)-mediated response or following electrical stimulation in small arteries [23,24]. This conducted hyperpolarization/dilation permits a rapid surge of blood flow which could be critical in pathophysiological conditions [16,23]. However, an understanding of the mechanism(s) underlying this conductive hyperpolarization/dilation remains incomplete [25,26]. We hypothesized that the all-or-none-like activation/deactivation of Kir channels underlying the bimodal RP plays an important role in conducted hyperpolarization/dilation [27].

It remains unclear whether a bimodal RP distribution generally exists in other vascular beds, besides the SMA [16]. A bimodal RP was reported in coronal arterioles [28] prior to our characterization in the SMA [16]. Other reported mean RPs of VSMCs varied from -40 to -75 mV in various vessel preparations and recording methods [18,20,21,22,29,30,31], yet these data may also reflect an inherent functional heterogeneity among the vascular beds. Therefore, a careful comparison of electrical membrane properties of different vascular beds in the same conditions was undertaken in this study to determine whether bimodal RPs are unique to coronal and inner ear arterioles or present in other vascular beds. We report here that arterioles from the mesentery and brain pia have a very different RP distribution from that of the SMA (Fig 1*A*).

How exactly the Kir current integrates with other ion currents to yield the bimodal RP distribution remains to be clarified. For instance, with the qualitative knowledge of Kir unique feature of the regenerative activation (disinhibition) and inactivation (inhibition) in mind, it is a puzzle why a moderate hyperpolarization of low RP cells triggers a large regenerative hyperpolarization only in some but not all cells, and likewise in high RP cells, a moderate depolarization induced a big regenerative depolarization also in a portion but not all the cells [16].

In addition, inwardly rectifying Kir2.X channels contribute to the setting and maintenance of the RP in many types of cells including the VSMC [32,33,34]. The homomeric channels’ currents of the four isoforms (Kir2.1—Kir2.4) all show similar strong inward rectifying properties but their individual sensitivity to Ba^2+^ block differs significantly [35]. Heteromeric channels may show current properties and/or Ba^2+^-sensitivity similar to native Kir channels [36,37]. The four isoforms of the Kir2.X family are all expressed in human aortic endothelial cells (EC) [38] but apparently only Kir2.1 in rat arterial VSMCs [33]. With these considerations, we hypothesized either quantitative and/or qualitative differences in the expression of Kir2.X isoforms underlie the different RP distributions in the three vessels.

## Materials and Methods

### Animals and the arteriole preparations

Guinea pigs (Albino, 250–500g) were exsanguinated after induction of deep anesthesia with intramuscular injection (1ml/kg) of a mixture containing ketamine 500 mg, xylazine 20 mg and acepromazine 10 mg in 8.5 ml H_2_O. The SMA and the mesenteric (MA) and brain arteries (BA, the anterior inferior cerebellar artery) with arteriolar branches were rapidly excised. All vessels tested were under 100 *μ*m in diameter, and histological examination showed a single layer of smooth muscle cells, thus classified as arterioles. The vascular preparation was incubated in Krebs solution (see below) for 0.5 to 4 h prior to transfer to a recording bath chamber. After removal of spongy connective tissue, the SMA, or a similarly-sized branch of the BA or MA, were cut into 1.0–2.5 mm long segments, pinned with minimal stretch to a silicon rubber layer at the bottom of an organ bath (volume 0.5 ml) and continuously superfused with a 35°C Krebs solution composed of (in mM): NaCl 125, KCl 5, CaCl_2_ 1.6, MgCl_2_ 1.2, NaH_2_PO_4_ 1.2, NaHCO_3_ 18, glucose 8.2, and saturated with 95% O_2_ and 5% CO_2_ at 35°C (pH 7.4). These preparations were used for conventional intracellular recording or vaso-diameter tracking experiments. The animal use procedures were approved by the Oregon Health & Science University Institutional Animal Care and Use Committee.

### Intracellular recordings

As previously described [16,39], the microelectrode was filled with 2 M KCl with a tip resistance of 100–200 MΩ. Propidium iodide (1%) was added into the filling solution when needed for intracellular labeling (S1 Fig). Cells were impaled using a manual micromanipulator under a stereomicroscope. Transmembrane potentials and currents were simultaneously monitored by an Axoclamp 2B preamplifier (Axon Instruments, Inc. USA) or an npi SEC 10-LX preamplifier (npi Electronic, Tamin, Germany). The electrical signals were recorded on a PC computer equipped with Digidata 1322A & Axoscope9 using sampling intervals of 0.1, 0.5 or 10 ms, as needed. Known concentrations of drugs were applied via the bathing solution. The solution infusing the recording chamber could be switched, without change in flow rate or temperature, to one that contained a drug or one of different ionic composition. The initial RP of a cell was usually obtained 5 min after penetration as the RP stabilized and, in some cases, verified by electrode withdrawal. We did not distinguish between VSMCs and ECs except otherwise indicated in this study since these two cell types are gap junction-coupled and electrically behave similarly in arteriolar segments with functioning gap junctions [16,23,40], (also see S1 Fig).

### Tight-seal whole-cell recording

Whole-cell voltage-clamp recordings were obtained from dissociated arteriole cells or from cells *in situ* of short vessel segments as previously described [40,41]. Dissociated VSMCs and ECs were prepared from the SMA, and arteriolar branches of the BA and MA of guinea pigs. Arterioles were incubated for 20 min in a low-Ca^2+^ buffer solution containing (in mM): NaCl 142; KCl 5; CaCl_2_ 0.05; MgCl_2_ 1; Na-HEPES 4; HEPES 5 (pH 7.2), and glucose 7.5. Arterioles were then cut into ~1 mm long segments and digested for 20–25 min at 37°C with a buffer solution containing papain 1.5 mg ml^-1^, collagenase A 2 mg ml^-1^, bovine serum albumin (BSA) 3.75 mg ml^-1^ and DL-dithiothreitol 0.3 mg ml^-1^. After centrifugation (67 *g* for 5 min) and replacing supernatant with enzyme-free buffer 3 times, the preparation was triturated with a Pasteur pipette, and the cell-rich suspension transferred to a coverslip-bottomed Petri dish coated with poly-L-lysine (Sigma). Once the dissociated cells attached to the coverslip, the dish was mounted onto an inverted microscope (Zeiss, Axiovert 35) and superfused with normal extracellular solution (NES) composed of (in mM): NaCl 138, KCl 5, CaCl_2_ 1.6, MgCl_2_ 1.2, Na-HEPES 5, HEPES 6, Glucose 7.5, with pH 7.4 and osmolarity of 300 mOsm L^-1^ for whole-cell recording in room temperature. The VSMCs were identified by a characteristic spindle-shape (S2 Fig) [33,40,42]. The ECs were identified in the same dish by their oval shape with a diameter ≥ 8 μm combined with its characteristic membrane response: a hyperpolarization or an outward current response to 3 μM ACh, which was distinct from that of the VSMCs [40,41]. Sometimes, 5 to 10 ECs remained attached together as a tubule after trituration. These tubular ECs could be verified by their morphology using DIC microscopy [41].

For *in situ* recordings, short vessel segments (~0.4 mm long, 30–50 μm OD) were transferred to a glass-bottomed Petri dish filled with NES. The vessel was secured by platinum strips at each end, and digested with collagenase A (1.5 mg ml^-1^, Roche) dissolved in NES at 37°C for 15–30 min. After a complete wash-out of the enzyme, the dish was placed on the stage of the inverted microscope described above for the experiments.

Conventional or perforated patch whole-cell recordings were performed using an Axopatch 1D amplifier (Molecular Devices, USA). The pipette had a tip of ~1 μm OD and a resistance of 5–7 MΩ after being filled with a normal internal solution (NIS) containing (in mM): K^+^-gluconate 130, NaCl 10, CaCl_2_ 2.0, MgCl_2_ 1.2, HEPES 10, ethylene glycol-bis [β-aminoethylether] *N*,*N*′,*N*′-tetraacetic acid (EGTA) 5 (118 nM free Ca^2+^), and glucose 7.5, adjusted to pH 7.2 and osmolarity 290 mOsm L^-1^. For perforated patch recordings, the pipette was back-filled with a fresh solution of nystatin in NIS (200 μg ml^-1^). Pipette capacitance was fully compensated after a giga-seal formed. Membrane currents or voltage signals were low-pass filtered at 1 or 10 kHz (-3 dB); data were recorded on a computer equipped with a Digidata 1322A AD-interface and pClamp 9.2 software (Molecular Devices, USA) at a sampling interval of 10, 20 or 100 μs. A Minidigi digitizer and Axoscope 9.2 software were used to simultaneously carry out gap-free recording at a sampling interval of 50 ms. The liquid junction potential (LJP) of the electrodes filled with NIS was 14.3 mV in the NES bathing solution, calculated using pClamp9 software. The LJP value was used for on-line correction setting or off-line data analysis to correct membrane potential values from conventional whole-cell recordings [43]. The whole-cell holding current at -40 mV was not significantly different between the cells of conventional whole-cell group with LJP-correction and the perforated patch group (n = 20 and 15, p > 0.05), thus data from the two groups were merged together.

The transient current passing the membrane input capacitance (C_input_) was routinely uncompensated in order to monitor and calculate the access resistance (*R*_*a*_) and other membrane parameters online or off-line. The off-line calculation was done by exponential fit to the capacitive current transients and by the commonly used equations [40,44,45]. The C_input_ for *in situ* cells was calculated according to C = Q/V, where the charge (Q) was obtained by a 2 or 3 term-exponential fit to the current transient elicited by a voltage step (V, in mV)[41]. The voltage clamping error introduced by the current (*I*) passing the access resistance was corrected offline according to the equation *V*_*m*_ = *V*_*C*_—*I R*_*a*_ (in which *V*_*m*_ is the actual clamped membrane voltage and *V*_*c*_ is the apparent command voltage), except where noted otherwise.

### Vessel diameter tracking

**Fig 2:**
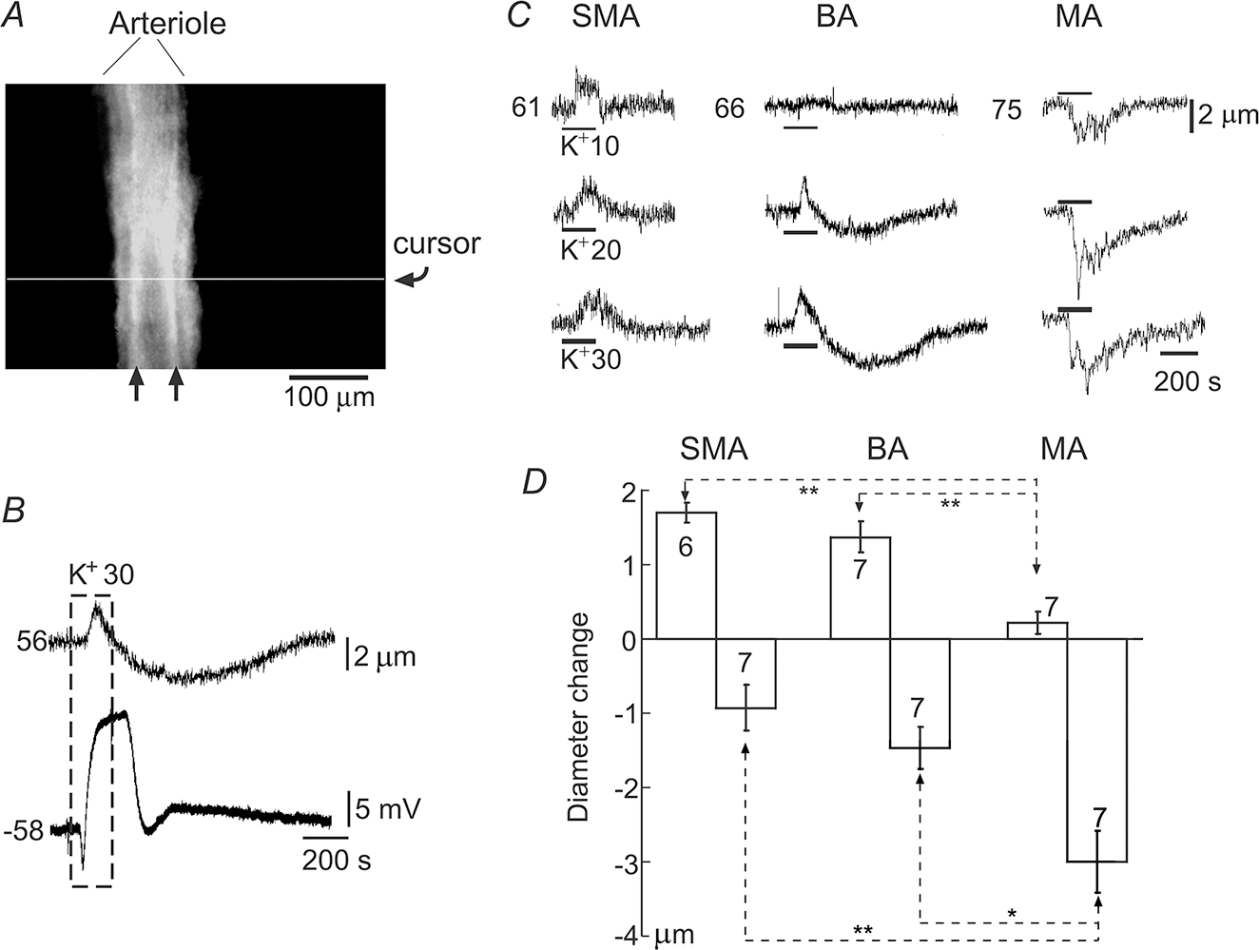
Vasotone responses to high K^+^ of the three arteriole types. (***A***) A video screen shot of a vessel segment undergoing diameter tracking. The horizontal line indicates the intensity sampling cursor. The outer-diameter of the vessel was determined at the muscle layer clearly seen (arrows) under dark-field illumination. (***B***) Simultaneous recordings of the vessel diameter (upper trace) and single cell membrane potential (lower trace) from an *in vitro* segment of a BA segment (56 μm thick). Superfusion of 30 mM K^**+**^ (boxed period, E_K_ = -39 mV) caused a brief small dilation (upward deflection in upper trace) followed by a lasting constriction; the same high K^**+**^ caused a multiphased potential deflection in this cell. (***C***) Representative traces showing diameter changes of the three type arteriolar segments to elevations in extracellular K^**+**^ as indicated (mM). The resting diameters (μm) are indicated to the left of each top traces. Note that the SMA showed largely a concentration-dependent dilation, the MA had concentration-dependent constrictions while the BA often showed biphasic responses. (***D***) Data plots of the diameter change in response to 20 mM K^**+**^, indicating that dilation or constriction prevailed in the SMA and MA, respectively, whereas biphasic response appeared most commonly in the BA. ** p < 0.01, * p < 0.05. The number of specimens tested is indicated in each column.

As previously described [15,46], segments of the SMA, BA or MA in the bath were dark field-illuminated by a fiber-optic lamp and imaged by a video camera (Sony XC-13) through a trinocular stereo-microscope (Fig 2*A*). Images of vertically-oriented arterioles were visualized on a monitor, recorded on tape and digitized by a video capture board (Matrox RainbowRunner Studio) using a Pentium III PC (1GHz). The digitized video signal was online processed by custom-made edge-detection software. Digitized vessel diameters (2–5 times/s) were saved to disks or converted on-line to analog signal and fed to Digidata 1322A AD-interface for simultaneous recording with the membrane voltage and current signals (Fig 2*B*).

### Immunofluorescence for Kir2.1

Albino guinea pigs were euthanized with an overdose of ketamine hydrochloride (100 mg/kg i.m.; Abbott Laboratories, USA) and xylazine hydrochloride (2 mg/kg i.m.; Phoenix Scientific, Inc., USA). After cardiovascular perfusion with saline followed by 4% paraformaldehyde, excised cochleae, BA and MA were immersed in the same fixative solution for 4 h. The SMA, BA and MA with arteriolar branches were isolated in 0.02 M PBS, pH 7.4, and then permeabilized in 0.5% Triton X-100 for 1 h. After immunoblock in 10% goat serum and 1% bovine serum albumin (BSA) in the PBS for 1 h, the specimens were incubated overnight in a solution containing anti-Kir2.1 antibody (rabbit polyclonal antibody, SC-28633, Santa Cruz Biotechnology, Inc., USA; 1:50 diluted with 1% BSA-PBS). Specimens were washed in 1% PBS for 30 min and incubated with secondary antibody Alexa Fluor 488 anti-rabbit IgG (1:100 diluted with 1% BSA-PBS; Invitrogen, USA) for 1 h. Then, 1 μg/ml propidium iodide (PI, Invitrogen, USA) was added to the solution for 10 min to label cell nuclei. After washing for 30 min, the vessels were mounted and observed on a Nikon Eclipse TE 300 inverted microscope equipped with a Bio-Rad MRC 1024 confocal laser scanning system (Bio-Rad, Hercules, CA). Negative controls were prepared by omitting primary antibodies. Fluorescence signals were imaged with 488-nm excitation and 520 nm emission filters. Cell nuclei images were taken with 568-nm excitation and 615 nm emission filters.

### Immunoblotting and RT-PCR analyses of Kir2.X gene expression

Arteriolar segments of SMA, BA, and MA were separately disrupted in lysis buffer containing (in mM): Tris [pH 7.4] 10, phenylmethylsulfonyl fluoride 0.1 and EDTA 0.1, in 1% SDS and 10 g/ml leupeptin. The samples were resolved by SDS-PAGE in 9% gel and transferred to polyvinylidene difluoride (PVDF) membrane (Millipore). The membrane was blocked with 5% non-fat dry milk in Tris-buffered saline containing 0.1% Tween 20 for 1 h and then incubated with rabbit anti-Kir2.1 antibody (1:200, Santa Cruz Biotechnology) or anti-α-actin antibody (1:500, Sigma) overnight at 4°C. After washing, the membrane was incubated with horseradish peroxidase (HRP)-conjugated goat anti-rabbit IgG secondary antibody for 1 h at room temperature, washed again, and protein detected by chemiluminescence using SuperSignal West Dura Extended Duration Substrate (Pierce). The blot was exposed on Kodak BioMax MR film, and quantified by Adobe PhotoShop using densitometer function. Western blot data showed slightly different levels of actin among the samples. To ensure that similar amounts of protein samples were used in SDS-polyacrylamide electrophoresis, the same membrane used for Western blot was stained with Coomassie blue after the protein detection. The membrane was destained and scanned for quantitative analysis. Band intensities of all protein bands in each lane were quantified and relative densitometric values obtained.

Total RNA from each tissue was isolated by using RNAqueous-Micro Kit (Ambion) according to the manufacturer’s instruction. First-strand cDNA was synthesized from 1 μg of total RNA in a 20 μl reaction mixture containing oligo(dT) to hybridize to 3’ poly(A) tails of mRNA, and SuperScript II Reverse Transcriptase (Invitrogen) to catalyze synthesis. RNA template was removed after cDNA synthesis, by digestion with RNase H. For RT-PCR, 1 or 3 μl of the reverse-transcribed cDNA was used in each reaction catalyzed by AmpliTaq DNA polymerase (Applied Biosystems). Primers used for PCR are:

gpKir2.1 (328 bp fragment)

5’-CCTCCCATTTCCACTGCGTGTGTCC-3’

5’-GCCAACTTCATGCCGTCCTCTTCC-3’

gpKir2.2 (374 bp fragment)

5’-CGCTGGATCAGATAGACATTGATG-3’

5’-CAAGAGAAACTTGTTCTCCACCAG-3’

gpKir2.3 (324 bp fragment),

5’-TACTCACGCTTCCACAAGACCTAC-3’

5’-CCTGCGGTAGGAGATATTGTCTAG-3’

gpKir2.4 (368 bp fragment)

5’-ATGAGATCGACTCTGCCAGTCCTC-3’

5’-GCAGCTGAGAGCAAGCTCATTCTC-3’

gpα-actin (388 bp fragment)

5’-TCTGGCACCACTCTTTCTACAATG-3’

5’-TTGATGTCCCGCACAATCTCACGC-3’

The RT-PCR was performed in an Applied Biosystems thermal cycler with the following conditions: 95°C for 2 min followed by 40 cycles of 95°C for 30 sec, 57°C for 30 sec, and 72°C for 1 min for all reactions. PCR products were analyzed by electrophoresis in 2% agarose gel.

### Statistics

Data statistics were normally shown as mean ± SE of the mean. Student’s *t*-test was normally used to determine the significance of the difference using software KaleidaGraph 3.6 or Microsoft Office 2007 Excel (USA) unless otherwise stated.

## Results

### Distinct bimodal RP distributions in the SMA, BA and MA

Conventional intracellular recordings from cells in the SMA, arteriolar branches of BA (anterior inferior cerebellum artery) or MA revealed RPs of -55 ± 0.77, -66 ± 1.5 and -68 ± 1.1 mV (Fig 1A, n = 573, 88 and 84), respectively. The mean RP of the SMA was significantly less negative than the BA or MA (p < 0.001), without any statistically significant difference between the latter two vessels (p = 0.38). Cytological identification by PI-labeling revealed that electrophysiologically-recorded cells included both the VSMC and EC in a roughly 1:1 ratio in all vessels (S1 Fig, also see [24,39]). The passive membrane properties of the SMC and EC were apparently similar due to gap junction coupling between these cell types [16,39]. We henceforth do not distinguish these two types of cell, unless otherwise stated.

As previously reported [16], the RP of this pool of SMA cells (n = 573) showed a prominent bimodal distribution (Fig 1*A*). Modeling with a mixture of two Gaussian functions revealed two peaks at -76 mV (high RP) and -40 mV (low RP) respectively with about equal counts and a trough at ~-60 mV (also see [16]).

In contrast, the RPs of BA and MA cells exhibited a very different bimodal distribution (Fig 1*A*), with reduced counts of low RP cells (BA) and a shallower trough (MA) than for the SMA. The high RP level of the SMA and BA (-76 and -77 mV) at the peak frequency is significantly more negative than that of the MA (-71 mV, p < 0.01, likelihood ratio test, [47]. The low RP level (-40 mV) of the SMA at the peak frequency is significantly less negative than that of the BA and the MA (-51 and -52 mV, respectively, p < 0.01). When we used -60 mV as a convenient boundary, cells could be roughly divided into two groups [16]: the high RP cells (≤-60 mV) and the low RP cells (≥ -60 mV). The low RP/high RP cell ratio was 1.27, 0.44 and 0.24 (n = 321/252, 27/61 and 16/68) for the SMA, BA and MA, respectively (with average vessel outer diameter of 33 ± 3.8, 58 ± 6.7 and 74 ± 9.6 μm; n = 65, 54 and 46). The ratio difference was statistically significant (p < 0.001, χ^2^) between the SMA and the BA or MA, but not between the BA and MA (P > 0.05).

Taken together, the three arteriole types display apparently distinct RP distributions with significant differences in RP means and low/high RP cell ratios, which explained the different vasotone in response to K^+^ elevations (see below).

### Comparison of membrane potential and vascular tone responses to high K^+^

A moderate elevation of extracellular K^+^ from typical 5 mM up to 20 mM often dilates, and higher elevations constricts, small arteries, which may play a key role in regulation of microcirculation [16,17]. We sought to combine these observations with RP and vasotone measurements and compare the three arteriolar beds.

A moderate elevation of extracellular K^+^ always reversibly depolarized high RP cells (~-75 mV), and typically hyperpolarized low RP cells (~-40 mV) in all three vessels (Fig 1*B*). The hyperpolarization was often reversible upon washing with normal bath solution. Occasionally, especially (~10%) in the SMA and BA, the RP might shift to a high RP with no recover to its initial low RP level (see Fig 6B and Fig 7A of ref. [16]). Fig 1*C* depicts the plots of cells randomly sampled from the SMA, BA and MA. A linear regression of the plots gave a gross estimation of the macro-response for a vessel type. A comparison of the linear regression indicated that 10 mM K^+^ predominantly depolarized MA vessels, yet hyperpolarized or depolarized cells in the SMA and the BA according to their original low or high RP, respectively. As a vasotone response is expected resulting from integration of individual cellular responses, these regression lines of depolarization-hyperpolarization predict that 10 mM K^+^ would predominately induce a constriction in the MA and a biphasic or a variable small response in the SMA and BA.

Elevation of extracellular K^+^ induced differing vascular tone responses in the three vessels (Fig 2). Superfusion with 10 mM K^+^ generally dilated the diameter of the SMA or BA by 1–2 μm (or 2–5%), and constricted the MA by 2–7 μm (3–9%). Superfusion with 20 or 30 mM K^+^ often induced a biphasic response: a transient dilation followed by a lasting constriction in all three vessels, although in the MA the dilation was small or absent. The dilation induced by 20 mM K^+^ in the SMA was larger than that of the BA but without statistical significance (Fig 2*D*). The 20 mM K^+^-induced constriction in the SMA was slightly smaller than that of the BA (p > 0.05) while both were significantly smaller than the MA. In the presence of 100 μM Ba^2+^, high K^+^-induced dilation was abolished in all vessels tested (n > 3 for each vessel, not shown) as frequently reported elsewhere (e.g., [48]), indicating the dependency of the dilation on a Kir current.

Simultaneous recording of diameter changes with single cell membrane potential was conducted in the three vessels with limited success (n = 2–5 for each vessel). Consistent with data presented above, 10 mM K^+^ hyperpolarized low RP cells associated with a small and fast dilation in the SMA and BA (n = 2 and 3, respectively). Interestingly, 20 and 30 mM K^+^ applications caused biphasic potential changes in low and intermediate RP cells associated with a biphasic vasotone change: a fast transient dilation followed by a longer lasting constriction in both SMA and BA (Fig 2*B*). The induced dilation and constriction lagged the initiation of induced hyperpolarization and depolarization, respectively, by about 1 min. For the MA, a depolarization in high RP cells was associated with a constriction in both 10 and 30 mM K^+^ application (n = 3).

Taken together, the above data demonstrate that the moderate high K^+^-induced potential and vasotone response patterns are closely related to the population distribution of RPs, i.e., vessels with mostly high RP cells generally depolarize and constrict; and vessels with predominantly low RP cells will hyperpolarize and dilate; while vessels with approximately equal proportions of cells with high and low RPs will have a biphasic vasotone response (a fast transient dilation followed by a longer lasting constriction).

### Barium sensitivity of membrane potentials of the three vessels

**Fig 3:**
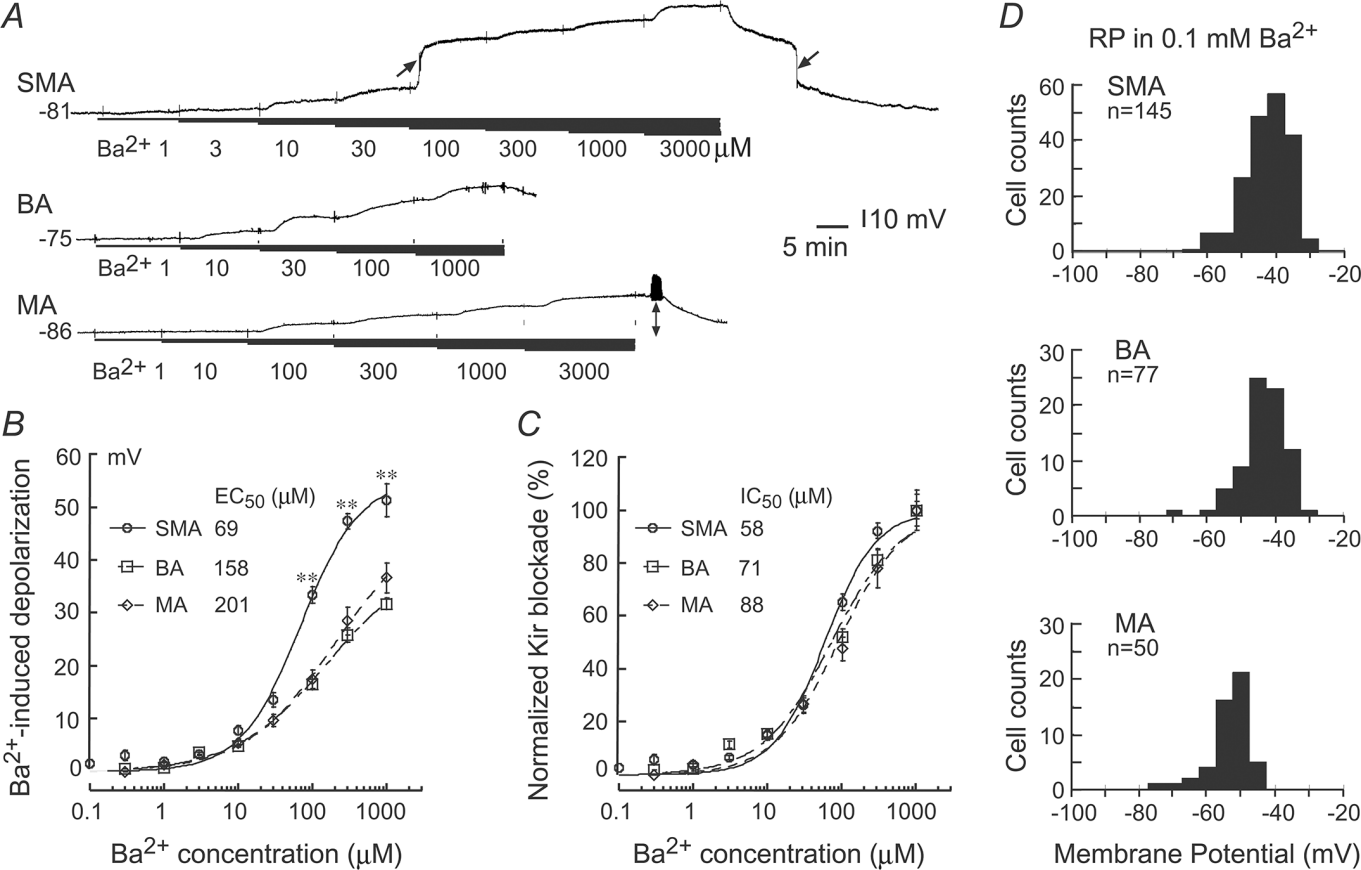
Ba^2+^ induces concentration-dependent depolarization of the three vessel types. (***A***) representative traces of incremental depolarization induced by increased Ba^**2+**^ concentrations in high RP cells of the SMA, BA and MA. Note that 100 μM Ba^**2+**^ caused a rapid large depolarization in this SMA cell, and a rapid repolarization upon washout (arrows on top trace). The dual-headed arrow denotes action potentials in the MA cell. (***B***) Hill equation fits to Ba^**2+**^ concentration-responses of sampled high RP cells (n = 7–25 for each data point) revealed a significantly lower EC_50_ in the SMA (p < 0.05) than in the BA and MA. The ** symbols indicate P < 0.01, compared to the other vessel types. The RP of sampled cells were -77 ± 1.07, 75 ± 1.01 and 75 ± 1.46, respectively (p > 0.05, n ≥ 30). (***C***) Using 1 mM Ba^**2+**^ depolarization as 100% Kir blockade standard, normalization of the data of ***B*** and Hill fit (see text) resulted in EC_50_s with no statistical difference (p > 0.05) among the three vessel types. (***D***) In 0.1 mM Ba^**2+**^, membrane potentials of the three types of arterioles all exhibited a single peak distribution. Of note, the mean and distribution peak of the MA were more negative than those of the SMA and BA (p < 0.05).

We hypothesized that the different bimodal distributions of the three vessels was mainly due to a differential Kir expression, since application of a Kir channel blocker, 0.1 mM Ba^2+^, caused a single-peaked distribution around the low RP level in all the three arterioles types (Fig 3*D*) (also see [16]).

Low micromolar Ba^2+^ is considered a selective blocker for vascular Kir channels, and is useful tool for studying Kir currents in isolation [17,49,50]. We measured the Ba^2+^ sensitivity of membrane potentials and whole-cell currents of these vessel types. Fig 3*A* shows typical membrane potential responses to incremental increases in extracellular Ba^2+^ in high RP cells where Kir is thought to be the dominating resting conductance. Notably, in the SMA, 10–100 μM Ba^2+^ frequently (8 out of 10) caused an extra-ordinal, fast and large depolarization (top trace in Fig 3*A*), which was present in less than half of the cells in the BA (2 out of 10) and only occasionally in the MA (1 out of 30). This phenomenon has been interpreted as a regenerative deactivation of the Kir channel due to its unique voltage dependency [16]. The EC_50_ based on Ba^2+^-induced depolarization (Fig 3*B*, data including cells with both typical and extra-ordinal depolarizations) showed a significantly smaller value in the SMA than those of the BA and MA, which was apparently problematic as the Hill fit curves were largely unsaturated at 1 mM Ba^2+^ and thus the EC_50_s were strongly influenced by the extrapolated depolarizations at >1 mM Ba^2+^. It is known that mM and higher concentrations of Ba^2+^ become increasingly less specific for Kir, i.e., it also blocks K_V_ (voltage-gated potassium channel) and BK_Ca_ (big conductance calcium-activated potassium channel) channels [17]. To minimize the influence of the non-specific effects, we set 1 mM Ba^2+^-depolarization as 100% Kir blockade for data normalization and the upper limit of Hill fit (Fig 3*C*), which revealed EC_50_s of 58, 71 and 88 μM for the SMA, BA and MA, respectively (p > 0.05).

Considering Ba^2+^ concentrations higher than 100 μM are less Kir selective and Ba^2+^-sensitivity of Kir channels is voltage-dependent, more potent at deeply hyperpolarized potentials [49], and thus the Hill fit in Fig 3*B* and 3*C* is complex to interpret; these IC_50_s cannot be regarded as accurate measurements of Kir channel sensitivity to Ba^2+^ blockage, a further rigorous whole-cell voltage clamp investigation was conducted.

### Whole-cell Kir current and Ba^2+^-sensitivity in the three vessels

**Fig 4:**
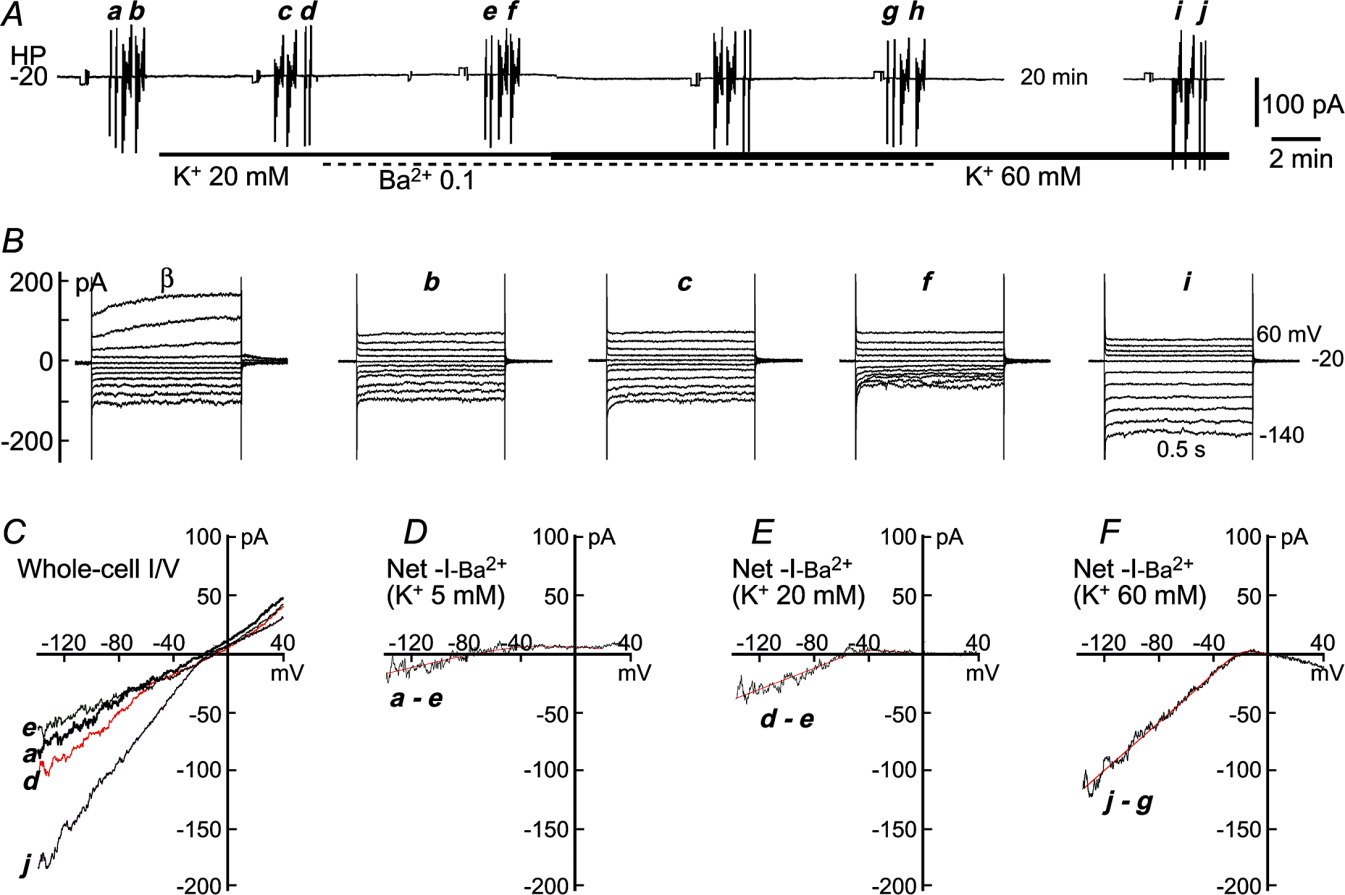
Measurement of Kir parameters at 3 extracellular K^+^-concentrations of a dispersed SMA smooth muscle cell. **(*A***) Gap-free trace of holding current (HC) at -20 mV, depicting the experimental protocol. The vertical deflections were caused by repeated ramp commands (***a*, *d*, *e*, *g*, *j***) and step commands (***b*, *c*, *f*, *h*, *i***) for construction of I/V curves in three K^**+**^ concentrations and 0.1 mM Ba^**2+**^. A cocktail of 1 mM 4-AP and TEA, 1 μM nitrendipine and 3 μM glipizide was used throughout the trace to suppress the background Ca^**2+**^-current and the K^**+**^-currents other than Kir. (***B***) Step-induced whole-cell currents revealed a mild inward rectification (Kir) in control (***B*.*b***, 5 mM K^**+**^), which was enhanced by 20 mM and 60 mM K^**+**^ (***c*** and ***i***, respectively) whereas suppressed by added 0.1 mM Ba^**2+**^ (*B*.*f*). Traces ***B*.β** were recorded prior to adding of the cocktail, showing that the delayed outward rectifier K^**+**^-currents (K_V_ and BK_Ca_) but not Kir was suppressed by the cocktail (***B*.β vs. *B*.*b*)**. (***C***) Ramp-constructed whole-cell I/V curves in 5, 20 and 60 mM K^**+**^ (***a; d*, *j;*** E_K_ ***=*** -83; -48; -20 mV) and in added 0.1 mM Ba^**2+**^ (***e*, *g; g*** *was* removed for clarity because they were overlapped), depicting the same results as in ***B***. (***D-F***) The I/V curves of the net Ba^**2+**^-sensitive current by the subtractions indicated below the curve. The overlapping smooth curves are the least square fit to the I/Vs (between -140 and 0 mV) with the Boltzmann function, $I_{Kir}=(V_{m}-V_{r-Kir})*G_{Kir}$, where $G_{Kir}=G_{\text{max}}/(1+\text{exp}((V_{m}-V_{0.5})/k))$, which revealed Kir parameters as G_max_ = 270 pS, V_0.5_ = -59 mV, a slope factor *k* = 30 mV/e-fold in 5 mM K^+^ (*D*). In 20 and 60 mM K^+^ (***E*** and ***F***), G_max_ = 447 and 862 pS; V_0.5_ = -42 and -22 mV; and *k* = 8.9 and 7.3 mV/e-fold, respectively. The cell had an input capacitance C_m_ of 11 pF. All the traces in ***B*** and I/V curves in ***C*** were averages of two repeats (see *A*).

**Fig 5:**
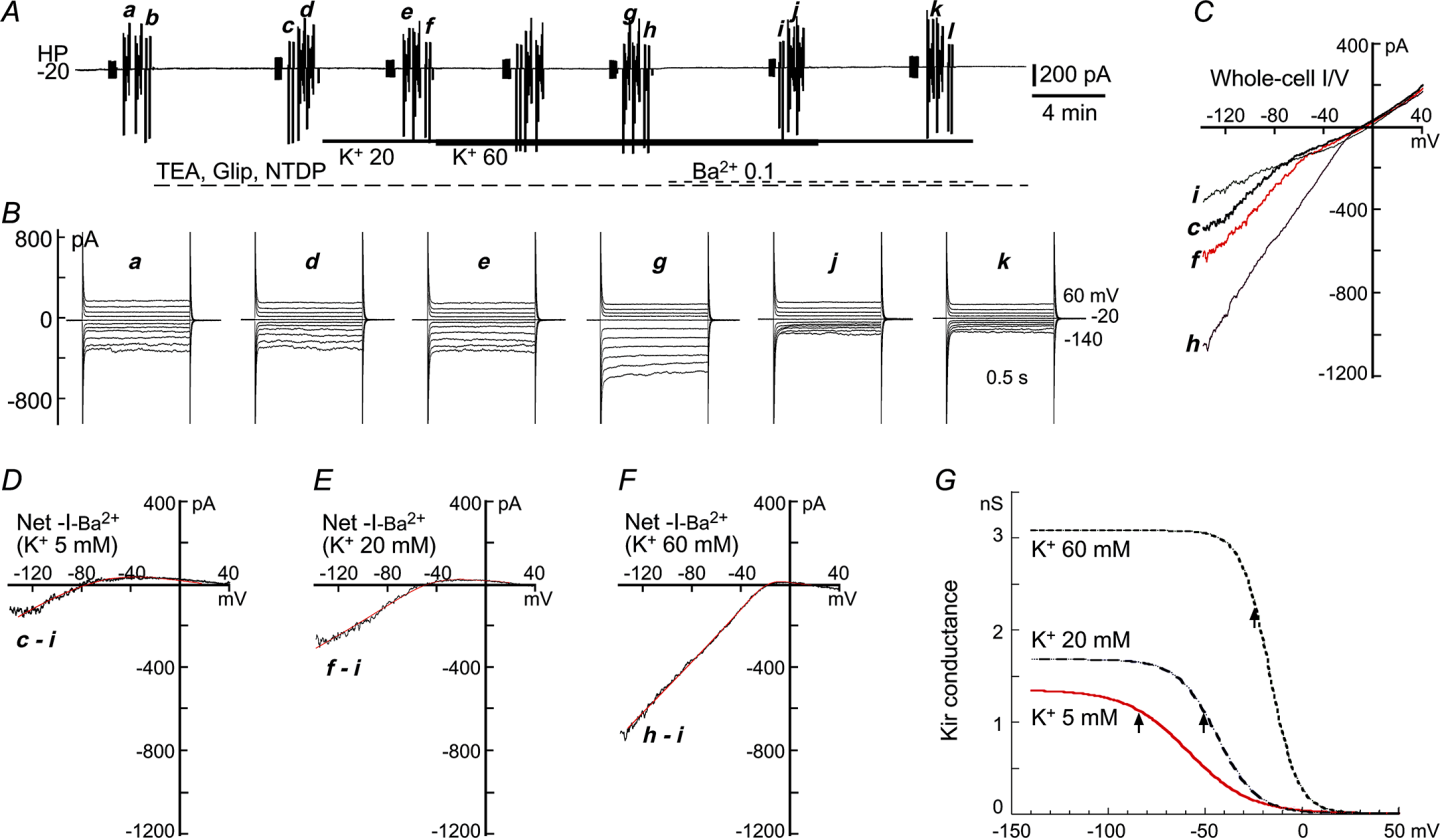
Measurement of Kir parameters at 3 extracellular K^+^-concentrations for SMA ECs. (***A***) Chart trace of HC at -20 mV, showing the experimental protocol. The vertical deflections were caused by repeated ramp commands (***b*, *c*, *f*, *h*, *i*, *l***) and step commands (***a*, *d*, *e*, *g*, *j*, *k***) for construction of I/V curves. (***B***) Step-induced whole-cell currents revealed a significant inward rectification in control (***B*.*a***, 5 mM K^**+**^), which was not affected by the cocktail solution (***B*.*d***) but enhanced by 20 mM and 60 mM K^**+**^ (***e*** and ***g***, respectively), and suppressed by added 0.1 mM Ba^**2+**^ (***B*.*j*** and ***B*.*k***). (***C***) Ramp-constructed whole-cell I/V curves in 5, 20 and 60 mM K^**+**^ (***c*, *f*, *h***) and in added 0.1 mM Ba^**2+**^ (***i*,*l; l*** was removed for clarity), depicting the same results as in ***B***. (***D-F***) The I/V curves of the net Ba^**2+**^-sensitive currents in the three [K^**+**^]_o_s. The overlapping smooth curves are the least square fit of Boltzmann function to I/Vs (see Fig 5 legend). The curve fitting revealed Kir parameters as G_max_ = 1.34 nS, V_0.5_ = -59 mV, a slope factor *k* = 15.9 mV/e-fold in 5 mM K^**+**^ (***D***). In 20 and 60 mM K^**+**^ (***E***, ***F***), G_max_ = 1.67 and 3.07 nS; V_0.5_ = -44 and -17 mV; and *k* = 10.2 and 7.2 mV/e-fold, respectively. (***G***) Voltage-dependency plot of the Kir conductance in the three [K^**+**^]_o_s. Arrows (**↑**) denote the E_K_, respectively. The recorded cell was located within a short EC tubule having an input capacitance C_m_ of 78 pF and the capacitive transient was fitted with a single term exponential function, indicating tight electrical coupling of multiple ECs (also see [41])

Whole-cell voltage-clamp recordings were conducted in more than 100 cells from each vessel that were dissociated or *in situ* within a short arteriolar segment; in the latter cases, the cells were less enzyme- and trituration-treated (e.g., Fig 4 vs. Fig 5, also see [40,41]. The general membrane properties of these cells were similar to those published previously (Table 1 in [41]). Briefly, *in situ* cells typically exhibited a low input resistance (~0.5 GΩ for the SMA and BA, ~0.3 GΩ for the MA) or higher input conductance, high input capacitance (about 70, 150 and 250 pF for the SMA, BA and MA), than dissociated cells (about 3.5, 3.5 and 3 GΩ; 6, 9 and 13 pF for the SMA, BA and MA, respectively), indicative of ubiquitous gap junction-coupling in the three arteriole beds but with tighter electrical coupling in the MA than the BA or SMA (S1 Fig). Gap junction-mediated electrical coupling was blocked by 30 μM 18β-glycyrrhetinic acid (18βGA), resulting in a complete electrical isolation of the recorded cells in all three vessel types [40,41].

Whole-cell I/V curves of visually identified VSMCs exhibited a robust TEA/4-AP-sensitive outward rectification with (S2 Fig, Fig 4B.β.) or without (S3 Fig) a robust Ba^2+^-sensitive inward rectification, whereas ECs had a mild or no such outward rectification with mostly a robust inward rectification (Fig 5, also see [41]). These property differences between the VSMCs and ECs were mainly observed in SMA cells (n = 40 and 10, respectively) and appeared to be also true in the other two arterioles, based on limited successful recordings from ECs of the BA and MA (n = 4 and 2, respectively). Since vascular cells express a number of resting currents in addition to the Kir and the cells are not spherically shaped for ideal voltage-clamping, we routinely add a cocktail of blockers,4-AP, TEA, glipizide and nitrendipine [15,17,18,51] to minimize these background currents throughout the experiments thus to improve the space-clamping and the accuracy in measuring the Ba^2+^-sensitive Kir current (Figs 4 and 5).

**Fig 6:**
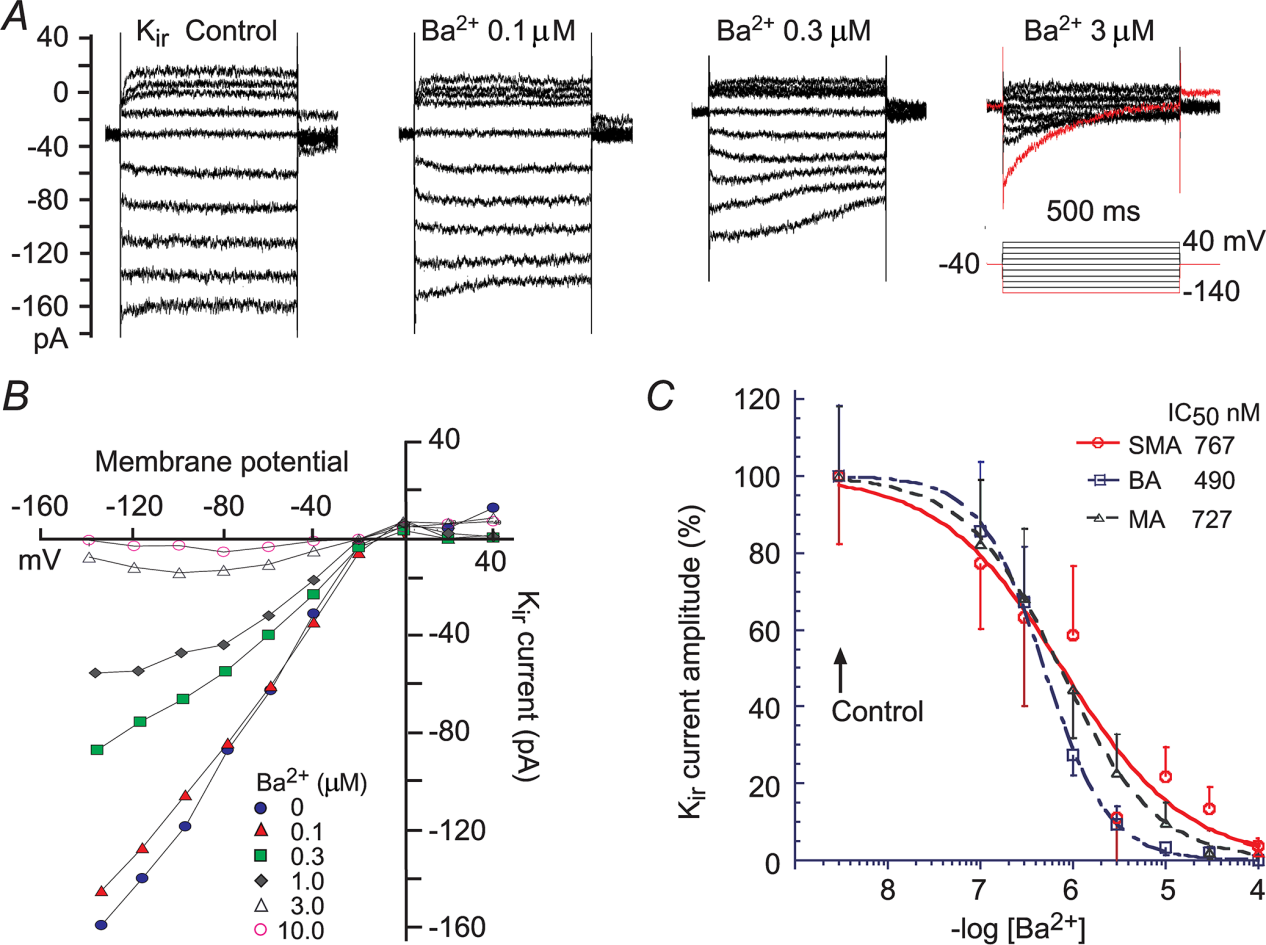
Ba^2+^ inhibits K_ir_ current in similar concentration-dependent manner in the three arteriole types. (***A***) Sample recordings of whole-cell net K_ir_ current in a BA cell by subtracting current traces in 0 mM K^**+**^ from those in 60 mM K^**+**^ (see S4 Fig). Note that the Ba^**2+**^ blockade on the inward current was concentration- and time-dependent (stronger block near the end of voltage step). All traces were an average of three sweep repeats. (***B***) I/V plot of Kir values near the end of each step in ***A***, showing that Ba^**2+**^ blocking was voltage-dependent (more effective at -100 mV and beyond than at -80 mV and less negative potentials). (***C***) Ba^**2+**^-concentration-inhibition (at -120 mV) plots of Kir current means from the three vessel types in this study. Hill equation curve fit revealed IC_50_s of 682, 498 and 768 nM for the SMA, BA and MA cells respectively (n = 2–12 for each data points). Hill number is near 1 for all fits. All cells were recorded *in situ* within a short arteriolar segment in the presence of 30 μM 18βGA, 1 mM TEA, 1 μM nitrendipine and 3 μM glipizide.

To compare Kir sensitivity to Ba^2+^ between the three vessel types, we isolated Kir currents by a method introduced by Xu et al. [52], i.e., net Kir component in a whole-cell I/V curve was obtained by subtracting the I/V curve recorded in K^+^-free solution (S5 Fig). Fig 6 shows the representative recordings of net Kir current in 60 mM [K^+^]_o_ and the effects of Ba^2+^ (0.1 to 100 μM). The results indicate that Ba^2+^ inhibition of the Kir current exhibited a time-dependent and voltage-dependent manner, consistent with previous reports (e.g., [49]: Ba^2+^ inhibition on the inward current becomes stronger towards the end of 0.5–1 s hyperpolarizing steps and at the deep hyperpolarizing voltages beyond -100 mV). At -120 mV, least square curve fit of Hill equation to the concentration-response plots revealed IC_50_ as 767, 490 and 727 nM in the SMA, BA and MA cells, respectively. The differences between any two IC_50_ values had no statistical significance (p > 0.05). Based on this result, 100 μM Ba^2+^ is expected to yield nearly 100% inhibition on Kir current in the three vessels. In other words, the net current sensitive to block by 100 μM Ba^2+^ could be regarded as the isolated Kir current in these vessels.

### Comparison of Kir biophysical parameters of the three arterioles

**Fig 7:**
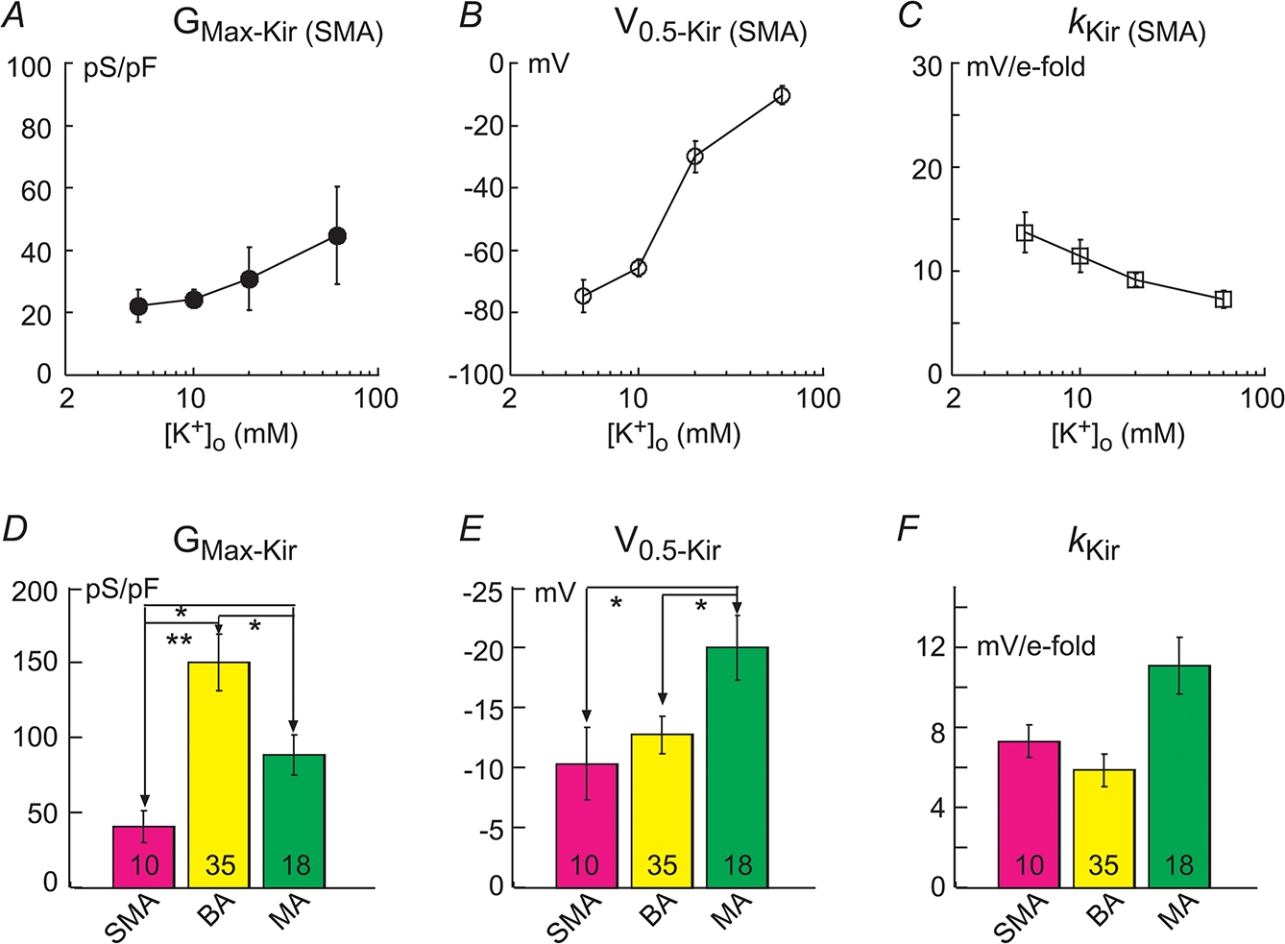
Kir parameters in SMA cells at four extracellular K^+^ concentrations and in cells of the 3 vascular types at 60 mM K^+^. Cells were randomly sampled and parameters obtained by the method shown in Figs 5 and 6. (***A-C***) Panels show that the maximal Kir conductance (G_Max- Kir_) tends to increase with the [K^**+**^]_o_ (***A***), the half-activation voltage (V_0.5-Kir_) tends toward depolarized potentials (***B***), while the slope factor (*k*_Kir_) becomes smaller (steeper), along with the [K^**+**^]_o_ elevation (***C***). n = 6–15 for each data point. (***D-F***) Panels show the G_Kir-Max_ density is significantly different in cells of the three arterioles in 60 mM [K^**+**^]_o_: BA>MA>SMA. MA cells appeared more negative V_0.5-Kir_ and less steep *k*_Kir_ than those of SMA and BA. * p < 0.05; ** p < 0.01. Data from high quality recordings of ECs/SMCs of 4/6, 2/33 and 0/18 in the SMA, BA and MA, respectively.

First, we characterized the biophysical parameters of the whole-cell Kir current at four extracellular K^+^ concentrations ([K^+^]_o_) by Boltzmann function fit to the 100 μM Ba^2+^-sensitive net current in SMA cells (Fig 7*A*–7*C*). With elevation of [K^+^]_o_ from 5 to 10, 20 and 60 mM, the maximal conductance (G_max-Kir_) increased, the half-activation voltage (V_0.5-Kir_) shifted to more depolarized level and the slope factor (*k*_-Kir_) became smaller (steeper in deactivation curve) (Fig 7*A*–7*C*). The same tests were repeated in the BA and MA (n = 2–5), and revealed similar trends.

Quantitative comparison of Kir parameters among the three vessels was conducted only in 60 mM [K^+^]_o_ (Fig 7*D*–7*F*), since the majority of cells recorded in 5–20 mM K^+^, especially in the SMA and MA, had small or negligible Kir currents. The results in Fig 7*D*–7*F* were derived from randomly recorded VSMCs and ECs with measurable Kir currents, with a EC/VSMC ratio of 4/36, 2/38 and 0/49 in the SMA, BA and MA, respectively. All recorded ECs had a robust Kir current at 60 mM [K^+^]_o_. However, 10 of 36, 36 of 38 and 25 of 49 of VSMCs had measurable Kir currents in the SMA, BA and MA, respectively. These results differ from a previous report that most VSMCs in small arteries have functional Kir [17] and from a report [53] that functional inwardly rectifying Ba^2+^-sensitive channels are restricted to the endothelial cell layer in the rat small mesenteric artery.

**Fig 8:**
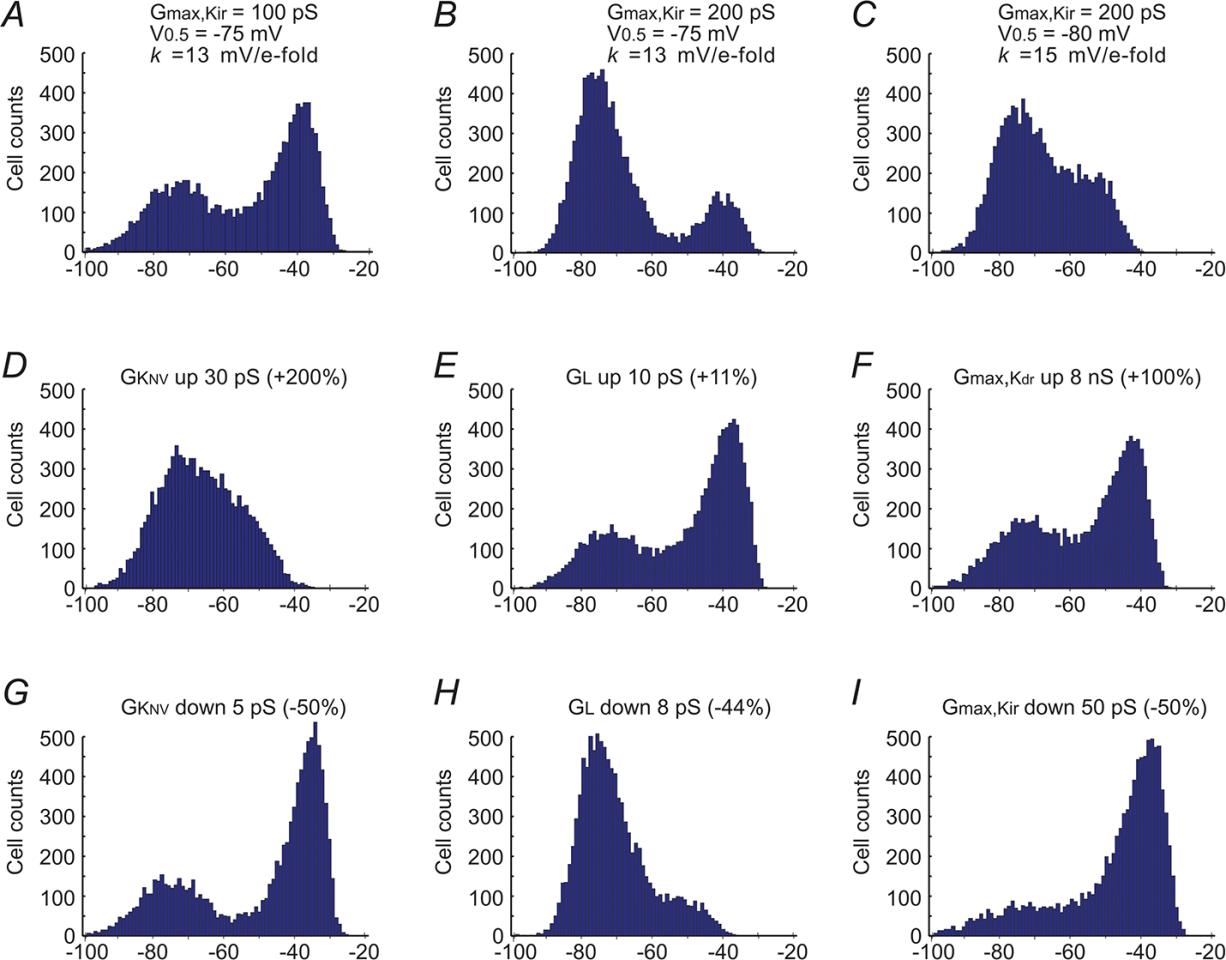
Computational modeling of the bimodal distribution of RPs and altered parameters of the resting ion channels on the distribution of RPs. Channels in this model included the 4 major RP-contributing conductances: the voltage-dependent inward rectifier K^**+**^ channel (Kir), the delayed outward rectifier channels (K_D_ or Kdr), and the non-voltage-dependent K^**+**^-channels (K_NV_) and leakage channels (L, see text). We computed the instantaneous conductances of Kir and K_D_ by Boltzmann equations, and the membrane potentials by the Hodgkin-Horowitz equation: $G_{Kir}=G_{\text{max},Kir}/\left(1+\text{exp}\left(\left(V_{m}-V_{0.5}\right)/k\right)\right)$; $G_{KD}=G_{\text{max},KD}-G_{\text{max},KD}/\left(1+\text{exp}\left(\left(V_{m}-V_{0.5}\right)/k\right)\right)$; $V_{\text{m}}=\left(E_{K}*\left(G_{Kir}+G_{KNV}+G_{KD}\right)+E_{L}*G_{L}\right)/\left(G_{Kir}+G_{KNV}+G_{KD}+G_{L}\right)$; Stable RPs were determined by programmed computation with MatLab software using up to 200 iterations after the initial parameters input (See S1 Dataset for Fig 8***A—*** 8***I***). (***A***) The initial parameter inputs included simultaneously the four conductances with randomly generated variations around the means derived from (rounded) whole-cell measurements of single SMA cells. Such inputs were repeated 10,000 times, resulting in a group RPs showing a bimodal distribution resembling that of the experimental RPs of the SMA (Fig 1***A***). (***B*** and ***C***) Change of the Kir parameters in ***A*** to mimic data in Fig **7*D*–7*E*** resulted in RP distributions that simulated the RP distributions of the BA and MA (Fig 1***A***). (***D-I***) RP distributions altered from ***A*** by increased and decreased G_KNV_ (***D***, ***G***), G_L_ (***E***, ***H***), increased G_max,KD_ (***F***) and decreased G_max,Kir_ (***I***). When G_max,Kir_ = 0, RPs always showed a single peak distribution regardless of any change in other conductances (not shown).

Data from Kir-current positive cells (Fig 7*D*–7*F*) indicate that the three vessel types have a significantly different G_max-Kir_ density: BA>MA>SMA; the MA cells have a more negative V_0.5,Kir_ (p < 0.05) and less steep slope factor (but p > 0.05). Regardless the reason(s) to cause these differences in current density and the voltage-dependency (e.g., difference in intracellular concentrations of polyamine [54]), when these parameter differences were used in computational modeling, the distinct RP distribution patterns of the three arterioles (Fig 1*A*) were largely simulated (Fig 8, see below).

### Role of Kir in bimodal RP distribution: computational modeling

To better understand the role of Kir in RP distribution, we employed computational modeling of the distribution based on a simplified ion channel composition in the whole-cell I/V relation, i.e., the RP is determined by the sum of resting (persistent) currents via four kinds of channels: inward rectifier K^+^ channels (Kir), delayed outward rectifier K^+^-channels (K_D_, including Kv and BK_Ca_), non-voltage-dependent K^+^-channels (K_NV_, including K_ATP_, small conductance calcium-activated potassium channel etc.), and the leakage channels (L, including non-selective cation channels, Cl^-^ channels etc.). We approximate the K_NV_ and L conductances as constant values within the typical range of RPs whereas the Kir and K_D_ conductances related to the membrane potential (V_m_) are described by Boltzmann functions:

$$G_{Kir}=G_{\text{max}-Kir}/\left(1+\text{exp}\left(\left(V_{m}-V_{0.5-Kir}\right)/k_{-Kir}\right)\right)$$ (1)

$$G_{KD}=G_{\text{max}-KD}-G_{\text{max}-KD}/\left(1+\text{exp}\left(\left(V_{m}-V_{0.5-KD}\right)/k_{-KD}\right)\right)$$ (2)

The resting potential follows the Hodgkin-Horowitz equation, analogous to GHK voltage equation when net transmembrane current is zero [55,56]:

$$V_{\text{m}}=\left(E_{K}*\left(G_{Kir}+G_{KNV}+G_{KD}\right)+E_{L}*G_{L}\right)/\left(G_{Kir}+G_{KNV}+G_{KD}+G_{L}\right)$$ (3)

The values of maximal Kir conductances (G_max-Kir_), its half activation voltage (V_0.5_) and slope factor (*k*) values were 0.1 nS, -75 mV and 13 mV/e-fold, respectively, rounded from the means of Boltzmann fit measurements of the net current sensitive to 100 μM Ba^2+^ in the SMA (n = 30, zero Kir current cells included in G_max-Kir_; also see Figs 4, 5 and 7; and S1 Dataset—Program lines for Fig 8). The mean G_max-KD_ and its V_0.5_, *k* values were rounded as 4 nS, 40 mV and 12 mV/e-fold, based on Boltzmann fit to leakage current-subtracted I/V of dispersed SMCs (S4 Fig, n = 10). G_KNV_ and G_L_ were 0.01 and 0.018 nS, rounded from I/V measurements of net current sensitive to blockers of 3 μM glipizide+1 μM nitrendipine and 1 mM La^3+^+30 μM niflumic acid, respectively (n≥ 6) (see S1 Dataset). The calculated E_K_ = -86 mV and the approximated E_L_ = 0 mV [17,55].

Using MatLab software (MathWorks, USA), simultaneous input of mean conductances of these 4 channels 10,000 times with random variations on a standard deviation (SD; Fig 8 and S1 Dataset) generated a RP population that showed a bimodal distribution (Fig 8*A*), which mimicked the experimental RP data for the SMA (Fig 1*A*). Referencing to the Kir parameter differences among the three vessels (Fig 7*D*–7*F*), increased G_max-Kir_ input from 0.1 to 0.2 nS resulted in a RP distribution (Fig 8*B*) that simulated the RP distribution for the BA (Fig 1*A*), and a G_max-Kir_ increase from 0.1 to 0.2 nS along with its V_0.5_ shift from -75 mV to -80 mV and *k* changed from 13 to 15 mV/e-fold transformed the distribution to a pattern mimicking data for the MA (Figs 8*C* vs. 1*A*). Reducing the Kir conductance G_max-Kir_ from 0.1 to 0.05 nS almost eliminated the hyperpolarized RP peak (Fig 8*I*). A zero G_max-Kir,_ combined with any alteration of other channel parameters did not yield a bimodal pattern of RP distribution (not shown). These results indicate a critical role of the Kir current in determining the RP distribution patterns.

In addition, Fig 8*D*–8*H* demonstrate that solely increasing (Fig 8*D*–8*E*) and decreasing (Fig 8*G*–8*H*) G_KNV_ or G_L_ from those values in Fig 8*A* also altered the RP distribution pattern drastically, while doubling the G_max-KD_ value had only a small effect on the pattern (Fig 8*F* vs. 8*A*). This is consistent with that K_D_ channels has no or little activation in the negative voltage levels up to -40mV, while K_NV_ and L conductances contribute to membrane potentials in the whole voltage range of interest, and thus always affect the voltage-sensitive channel Kir.

### Kir in genesis of regenerative RP shift—computational modeling

**Fig 9:**
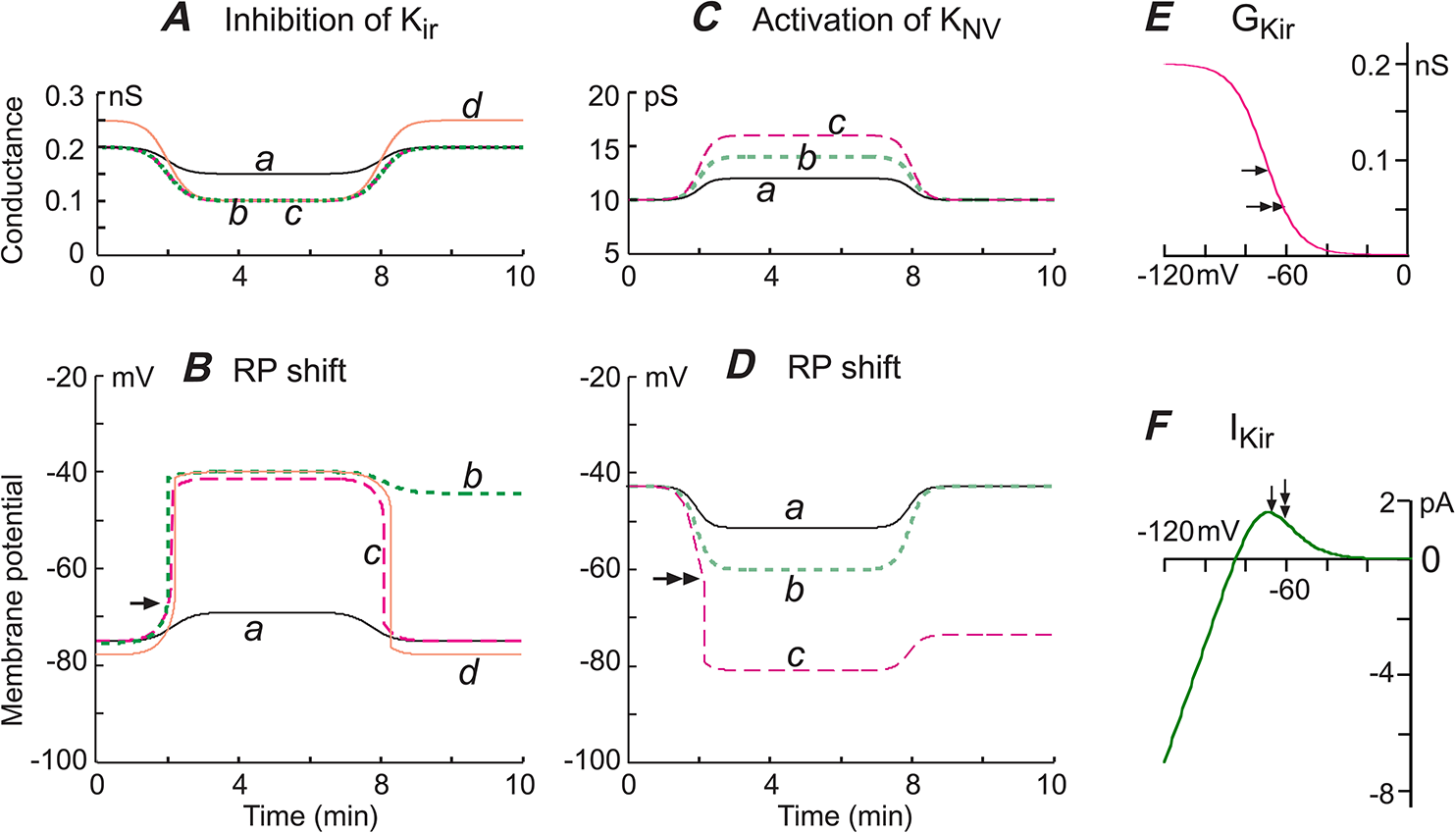
Simulating the time course of membrane potential shift by K^+^ conductance alterations. (***A***) The traces depict a sigmoidal reduction of G_max,Kir_ from 0.2 nS to 0.15 (***a***) and 0.1 nS (***b*, *c***) to simulate the inhibition of K_ir_ by wash-in and-out of increasing Ba^**2+**^ concentrations (***a & b*** with slope *k* = 8 mV/e-fold, ***c*** with *k* = 9 mV/e-fold); whereas ***d*** simulates another cell with higher control G_max,Kir_ (0.25 nS, *k* = 8 mV/e-fold). (***B***) The lower traces are calculated RP changes along with the G_max-Kir_ changes shown in ***A*** (***a-d***, respectively). Note that the K_ir_ inhibitions of ***b*, *c & d*** caused RP depolarization from a high to low level abruptly after a threshold (arrow) near -66 mV. Upon withdrawal of K_ir_ inhibition, the RP remained at low level (***b***) or returned to the control level (~-75 mV) due to bigger G_Kir_ slope factor (***c***) or bigger control G_max,Kir_ (***d***). Of note, ***A*.*c*** or ***A*.*d*** mimicked the Ba^**2+**^-induced potential shift observed in intracellular recording experiments (Fig 2***C*** of [16]. (***C***) Traces are plots of K_NV_ conductance changes from a control of 10 pS to 12, 16 and 16 pS, applied to the model to mimic NO-induced activation of K_ATP_ [64]. (***D***) The lower traces are calculated RP changes caused by the corresponding G_K,NV_ inputs (***a-c***). Note that the RP rapidly shifted from a low to a high RP level after reaching a threshold (double-headed arrow) near -62 mV when the G_K,NV_ increased to and beyond 16 pS (***c***) and the RP remained at high RP level after withdrawal of the large activations (***b***-***d***), analogous to an observation reported previously (Fig 7*C* of [16]). (***E & F*)** showing the threshold locations relative to the K_ir_ deactivation curve and I/V curve, respectively. The regenerative swift RP shift apparently occurs between these two arrows where the Kir deactivation curve shows a steep slope and the I/V curve shows a negative slope. Such regenerative RP shift was never observed when Kir conductance was nullified.

The voltage-dependence plots of Kir conductance in Fig 5*G* show that the conductance has a steep negative slope segment near its half-activation voltage (V_0.5_), e.g., ~-60 mV in 5 mM K^+^ normal external solution. Intuitively, within the steep slope domain, a small hyperpolarization would cause a regenerative process: *Hyperpolarization→more K*_*ir*_ *activation→K* ^*+*^ *efflux→more hyperpolarization*, and *vice versa*. However, the precise mechanisms, when or where such regeneration process occurs and the process is or isn’t reversible, are not well understood [16]. Using computational modeling of whole-cell data of typical Kir-expressing SMA cells in physiological solution (5 mM K^+^, e.g., Figs 4, 5 and 7), we analyzed this process (Fig 9 and S2 Dataset—Program lines for Fig 9).

In Fig 9, the series inputs of maximal Kir conductance (G_max,Kir_) were computer-generated with sigmoidal changes in simulation of Ba^2+^ wash-in and-out time course (Fig 9*A*, top traces). The dynamic RP were computed according to the equations above mentioned by a program in MatLab and the stabilized RPs plotted (Fig 9*B*, bottom traces). Fig 9*A* and 9*B* show that, in a cell initially with typical RP ~-75 mV, it takes >0.05 nS G_max,Kir_ reduction or >7 mV initial slow depolarization (threshold) to trig the rapid regenerative depolarization and thus a RP level shift (*b*, *c* in Fig 9*A* and 9*B*), which mimicked those observed in experiments (Fig 3*A*, also Fig 3*C* of [16]). On the other hand, the sub-threshold depolarization by a small Kir inhibition is always fully reversible upon withdrawal of the Kir inhibition (*a in* Fig 9*A* and 9*B*). Repeated alteration of various resting channel parameters indicated that the threshold level was determined mainly by the conductance ratio between Kir and I_L_, with less effect of K_NV_.

Moreover, the big RP level shift seen in Ba^2+^ experiments was reversible or nonreversible upon washout (e.g., Fig 3*A*, SMA, vs. Fig 8 of [16]). Both situations were reproduced by the computational modeling: when Kir slope factor (*k*) or G_max,Kir_ was increased, the induced high-to-low RP shift tends to be reversible (*c vs*. *b* or *d vs*. *b in* Fig 9*A* and 9*B*).

### Diverse molecular expression of Kir2.X in the arteriolar types

Our measurements of the membrane potential response to Ba^2+^ and the biophysics of the Kir current revealed some significant differences among the SMA, BA and MA, leading us to hypothesize that there is heterogeneous expression of Kir2 isoforms among these vessels.

**Fig 10:**
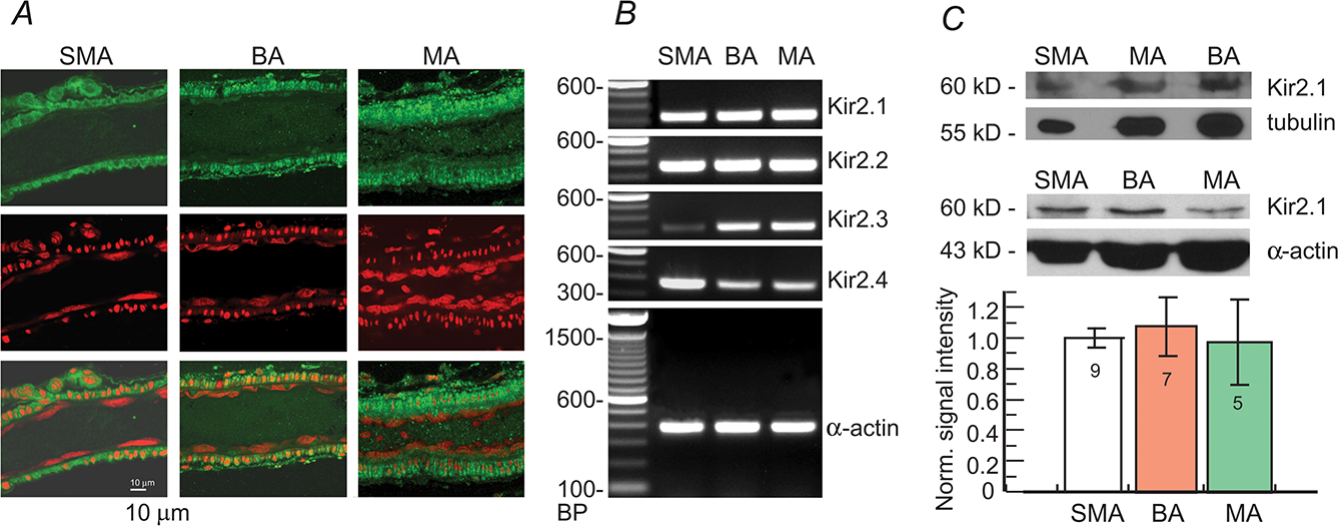
Molecular expression of Kir2.X channels in the three types of arterioles. **(*A***) Immunofluorescent localization of Kir2.1 channel proteins. Each panel is a single optical section of each arteriole. The top row shows Kir2.1 immunofluorescence (green), the middle row propidium iodide staining (PI, red, to label nuclei); and the bottom row is a merge of the two rows above. Kir2.1 is intensely expressed in the cytoplasm of VSMCs, with weaker Kir signals in the ECs indicated by their longitudinal nuclei. (***B***) RT-PCR of the four Kir2.X isoforms from the SMA, BA and MA revealed all four Kir2.X gene transcripts in each arteriole type, but at different expression levels: Kir2.1 and 2.2 were expressed at similar levels in all samples, Kir2.3 expression was higher in BA and MA than in SMA; and Kir2.4 exhibited higher expression in the SMA compared to BA and MA. α-Actin was used as an internal control. (***C***) Immunoblot analysis of Kir2.1 in the three arteriolar types with two different sample loading control (tubulin and α-actin). Top images are example blots giving an apparent lighter Kir2.1 band (but not statistically significant) in the MA compared with the SMA and BA. The column graphs show the means ± S.E. of signal intensity normalized with loading quantity within and across batches of experiments (n = 5–9). The density ratio of Kir2.1 was 1:1.07:0.97 of the SMA, BA and MA, respectively (p > 0.05).

Immunofluorescence revealed strong labeling for Kir2.1 antibody in all three arterioles (Fig 10*A*), suggesting that Kir2.1 is an essential isoform in these vessels. In single optical sections, intense fluorescence was localized in VSMCs, and was visibly weaker in ECs of all three arterioles. This appeared inconsistent to the findings above that all ECs exhibited a robust Kir current, while many VSMCs lacked significant Kir currents even in 60 mM extracellular K^+^. One interpretation is that the fluorescent signal for the Kir2.1 protein at the plasma membrane is too weak to be detected in ECs. A strong cytosolic Kir signal represents synthesized intracellular proteins, not functional channel proteins at the plasma membrane; the latter is obviously required for the Kir conductance. The apparently stronger Kir2.1 fluorescence in the MA muscle layer appears to be related to the higher density of cells.

To compare the relative transcript expression of the four Kir2.X isoforms in these arterioles (Fig 10*B*), we used RT-PCR. Transcripts for Kir2.1 and Kir2.2 are abundant in all three arterioles, Kir2.3 having the lowest and Kir2.4 the highest level in the SMA among the three vessels. Kir2.3 and Kir2.4 signals showed a gradient of SMA<<BA<MA and SMA>MA>BA, respectively.

Finally, immunoblot analysis of Kir2.1 expression in the three arterioles revealed that the protein specifically labeled by the Kir2.1 antibody was similarly expressed in the SMA, BA and MA (1:1.07:0.97, p > 0.05; Fig 10*C*), and was not correlated with the whole-cell current measurements of the three types arterioles (Fig 7). This suggests that the Kir2.1 proteins observed did not directly contribute to the different Kir current properties.

## Discussion

Using cellular electrophysiology, immunofluorescence and molecular approaches, we assessed the role of Kir channels in determining the membrane potentials of single cells and cell populations, and the vascular tone responses in three types of arterioles. Our primary findings are: 1) From intracellular recordings, the SMA showed a robust bimodal RP distribution with peaks at -40 and -76 mV, and significantly different from those of the BA and MA (Fig 1). The BA showed an apparent bimodal RP distribution but with substantially fewer low RP cells (or more high RP cells) than the SMA. The MA presented an asymmetric bimodal distribution with two peaks at -71 and 52 mV, and very few cells at RPs less negative than -40 mV. In the presence of 0.1 mM Ba^2+^, all the three vessels exhibited single-peak RP distribution around the low RP levels (Fig 3*D*). 2) Whole-cell measurement of the isolated Ba^2+^-sensitive Kir current with Boltzmann fit revealed that the maximum Kir conductance differs in these vessels: SMA<<BA>MA (Fig 7). 3) Computational modeling revealed that altering Kir current parameters alone, to parallel to the data of observed Kir currents from the SMA, BA and MA, can reproduce RP distribution patterns that simulate those of the three arterioles, respectively. Parameter alterations of non-voltage-dependent K^+^ channels and non-selective cation (or leak) channels also distinctively modify the modeled RP distribution. 4) We demonstrated, for the first time, the dynamic coupling of hyperpolarization/dilation and depolarization/constriction in arterioles.

Along with demonstrating that the individual cell RP level determines the high K^+^-induced membrane potential response, and that the population RPs determines the changes in vascular tone (Figs 1 and 2), this study, for the first time, quantitatively characterized the role of Kir channels in the diverse bimodal RP distributions and in vascular tone responses of different vascular beds. These findings provide a theoretical basis for better understanding of the conductive behavior of vascular dilation/constriction in small arteries/arterioles.

### High K^+^-induced changes in membrane potential and vasotonicity

Interstitial K^+^ elevation is thought to be a major factor in regulating blood flow in small arteries [16,57,58]. The different vasotone responses to high [K^+^]_o_ of the three arteriole types studied here are correlated to different population membrane potentials (Figs 1 and 2). Vascular cells can be roughly grouped into three: low RP (~-40 mV), high RP (~-75 mV) and intermediate RP (~-60 mV) cells [16]. Moderate increases in [K^+^]_o_ hyperpolarized low RP cells, likely due to extracellular K^+^ relief of Mg^2+^/polyamine block of the K_ir_ channel [16,59]. The increased K_ir_ conductance results in the membrane potential moving towards potassium equilibrium potential (E_K_) that is estimated to be more negative than the -67 and -49 mV potentials during 10 and 20 mM K^+^ exposures, respectively [16]. Therefore, [K^+^]_o_ elevation by ≤ 20 mM would hyperpolarize low RP cells, and depolarize high RP cells. In intermediate RP cells, high [K^+^]_o_ often causes a biphasic response consisting of an initial, transient hyperpolarization followed by a lasting depolarization, likely due to the fast change in K^+^-dependent Kir conductance and the slow kinetics of [K^+^]_o_ elevation during the superfusion of high K^+^ solution in our experiments.

In the SMA, more than half of cells had a low RP near -40 mV (Fig 1*A*) that allows activation of voltage-gated L-type Ca^2+^-channels and maintains Ca^2+^ influx and a vascular tone [18,60]. In these low RP cells, K^+^-induced hyperpolarization of these cells inactivates L-type channels, causing vessel dilation (Fig 2*C* and 2*D*). In high RP cells, a high K^+^-induced depolarization is expected to induce the opposite vasotone response—vasoconstriction. However, this latter response is usually masked by dilation (Fig 2*C* and 2*D*), because hyperpolarization prevails in the SMA (e.g., 10 mM K^+^ caused a mean of -7.1 mV hyperpolarization, Fig 1*A* and 1*C*). This interpretation is largely applicable to the SMA, and also the BA where the dilation was brief and followed by a constriction (Fig 2*B* and 2*C*). The reason for this biphasic response is likely due to more and more low-RP cells becoming high RP cells during high K^+^ exposure, and thus the high K^+^-induced depolarization and contraction in high RP VSMCs become dominating. In the MA, the majority of cells had a high RP ~-70 mV (Fig 1C), and exposure to 10 mM K^+^ depolarized cells by a mean of 9.8 mV. This is consistent with the observation that vasoconstriction was usually observed with elevated [K^+^]_o_ (Fig 2*C*).

### Role of Kir in bimodal RP distribution

Among RP-contributing channels, Kir is critical for a bimodal distribution of RP among groups of cells, as suggested by these, and our previous [16], studies. The decisive evidence is that a bimodal RP distribution does not occur when Kir conductance is absent or suppressed (e.g., by 100 μM Ba^2+^). The present study extended our understanding of how Kir contributes to a bimodal distribution quantitatively, in both static and dynamic dimensions.

In arteriolar cells, RPs are determined by multiple ion channel currents/conductance following the Goldman-Hodgkin-Katz equation or Hodgkin-Horowitz equation [56] and, to a minor degree (~5 mV), by the electrogenic Na^+^-K^+^-ATPase pump current [39,61]. Theoretically, alteration of all the RP-contributing channels and the electrogenic pump currents will change the RP distribution (Fig 8). In the RP range of most vascular cells (-30 to -80 mV), the RP-contributing conductances mainly include K_V_, BK_Ca_, Kir, ATP-sensitive K^+^-channels (K_ATP_), Cl^—^channels and non-selective cation channels [16,46,58,62,63]. These channel currents each affect the membrane potential in distinctive ways, which is briefly shown in Fig 8*D*–8*H*.

But of note, depolarization of an initially high RP cell to a low RP level (~-40 mV) in usually required ≤100 μM Ba^2+^ in the SMA and BA, but requires as much as 300 μM Ba^2+^ in the MA (Fig 3*A*), suggesting that an active K^+^ channel other than Kir with less sensitivity to Ba^2+^ may contribute to high level RPs in the MA (Fig 3*D*). It is also known that basal release of nitric oxide contributes 3–4 mV to the RP of the SMA by K_ATP_ [46,64]. With an enhanced or reduced K_ATP_ function, one can expect that the SMA would change its RP distribution pattern from Fig 8*A* to Fig 8*D* or Fig 8*G*, respectively. Nevertheless, a change of Kir parameters alone replicates the distinct RP distributions observed in these three arterioles, emphasizing the critical role of K_ir_ in setting the diverse RP distribution in physiological condition.

The unique voltage-dependency of the Kir conductance forms the biophysical basis of a regenerative abrupt RP shift (Figs 5 and 9). Using mathematical modeling of typical SMA cells in physiological solution, we depicted this process graphically. Fig 9 demonstrated that the abrupt RP shift takes a prior slow threshold depolarization to occur and *vice versa*. On the other hand, the sub-threshold depolarization or hyperpolarization is always fully reversible upon withdrawal of the resting channel alteration. It is conceivable that such regenerative shift makes RPs near -60 mV unstable and thus the cell counts troughed there, resulting in the bimodal distribution as we observed (Figs 1 and 8).

Moreover, the computational modeling also imitated the reversible and nonreversible big RP level shift seen in experiments. Briefly, in any given cell, a bigger Kir maximal conductance (relative to other resting conductances) with flatter slope factor makes the washout reverse more likely. In this regard, the MA cells have bigger *k* value than the SMA and BA cells (Fig 7*F*), which may have contributed to the MA’s very low counts of low RP cells (Fig 1).

Taken together, in arterioles, the bimodal RP results from the sum of K_ir_ and other membrane currents: generally the currents via other channels set a low RP background, while the K_ir_ current generates a high RP when the background reaches a negative level that promotes a regenerative K_ir_ activation (disinhibition), i.e., maximal K_ir_ activation underlies a high RP and deactivation of K_ir_ results in a low RP.

### Heterogeneity of Kir2.X expression

In contrast to a previous report that single smooth muscle cells of rat cerebral, coronary and mesenteric arteries expressed transcripts of Kir2.1, but not Kir2.2 and Kir2.3 [33], we detected Kir2.2, Kir2.3 and Kir2.4 transcripts in all three vessel types, as well as Kir2.1 (Fig 10*B*). The discrepancy is not fully understood but could be due to our tissues including both VSMCs and ECs, the latter of which almost ubiquitously exhibited the Kir current in this study. Based on the present findings, the Kir current in acutely isolated vascular cells, at least in the ECs could possibly be carried out by heteromeric Kir2.X channels.

When Kir2.1—Kir2.4 are individually expressed and form homomeric channels in *Xenopus* oocytes, the current sensitivity to Ba^2+^ block is significantly different among the isoforms [36]. Heteromeric channels formed by co-expression of Kir2.1/2.2, Kir2.1/2.3 and Kir2.3/2.4 in the oocytes all show a current similar to cardiac I_K1_ in both the sensitivity to Ba^2+^ block and kinetics [36]. We observed that the three arteriole types have similar Ba^2+^ sensitivity in their RPs and whole-cell Kir currents (Figs 3 and 6), suggesting that the three vessels have a similar molecular configuration of Kir2.X channels despite quantitative differences in expression of Kir2.3 and Kir2.4 transcripts (Fig 10*B*). The protein expression of Kir2.1 among the three vessels did not vary significantly (Fig 10*C*), and was not correlated with the Kir conductance density (pS/pF) of the three arterioles (BA>MA>SMA) (Fig 7), further suggesting that the abundance of channel proteins detected are likely nonfunctional. Therefore, the molecular mechanism(s) responsible for the distinct bimodal RP-distributions and its underlying diversity among the three types of arteriole in Kir conductance density and voltage dependency (Fig 7) require further investigation.

### Significance

Evoked focal hyperpolarization and dilation (e.g., by ACh, K^+^) can be conducted along a small artery beyond an exponential decay, permitting a fast increase in blood flow in response to a local physio-pathological release of K^+^ and other vasoactive factors. The present study advanced our knowledge on the mechanisms responsible for the conductive behavior of the vascular hyperpolarization/dilation [23,26].

The minimum biophysical requirements for this conduction, as for action potentials along a nerve or muscle fiber, may be summarized as: 1) electrical continuity along the conduction pathway, 2) regenerative swift shift between two states of membrane potential in cells, and 3) replenishment of the energy consumed during the membrane potential shift. This study also extends our previous data and hypotheses to a quantitative level, by showing that the regenerative activation and inactivation of the Kir current critically underlying the two RP states and the swift shift between these two states in the arteriole cells [16], which satisfies the second requirement. The other two requirements are also well met in the small artery/arterioles: gap junction-coupling among endothelial and smooth muscle cells and between these two cell layers ensures electrical continuity along the vessel [26,40]; and the ubiquitous Na^+^-K^+^-ATPase pump consumes ATP to restore the ion gradient changes caused by transmembrane ion fluxes during the shifts in RP.

We believe that such conduction-based fast blood flow regulation is carried out in most, if not all, tissues and organs, based on the ubiquitous presence of gap junctions, Na^+^-K^+^-ATPase pumps and the strong inwardly rectifying Kir channels/currents in cells of small arteries and arterioles. The observed RP distribution in the MA (Fig 1) appears to have dominating counts of high RP cells around -71 mV. However, it is known that cells in similar sized arterioles *in vivo* [22] or pressurized *in vitro* (60 mmHg [20]) normally show a RP ~-40 mV. It is likely that, *in vivo*, the MA will contain plenty of low RP cells because sheer stress will activate stretch-activated cation channels and depolarize cells [65]. The same trend of prevailing low RP cells is also expected in the BA and SMA *in vivo*. Therefore, a focal release of hyperpolarizing factor such as EDHF and K^+^ would normally trigger a conducted rapid and big hyperpolarization and dilation in small arteries in most, if not all, vascular beds.

## Supporting Information

S1 Dataset Computation program lines for Fig 8. (DOCX)

S2 Dataset Computation program lines for Fig 9. (DOC)

S1 Fig Dye coupling of the cells in the SMA and the arteriolar branches of BA and MA. Propidium iodide (PI) was used in electrode filling solution to label nucleus of the arteriolar cells. (**A** and **B)** Micrographs were taken with simultaneous fluorescent and DIC illumination to show the labeled cells and vessel contours of the SMA (A) and MA (B). A_1_ & B_1_ depict the intercellular dye-transfer between ECs, B_2_ between VSMCs, and B_3_ showing between EC and SMC. (**C)** is reconstruction of confocal images from a BA (AICA branch) showing EC-EC, SMC-SMC and SMC-EC dye-couplings. VSMC-VSMC (2/68) and VSMC-EC (1/68) dye-coupling were less frequently detected in the SMA than the other two vessel types.
(TIF)

S2 Fig Ba^2+^-sensitive Kir conductance in a dissociated SMA single smooth muscle cells. (***A***) Holding current trace with truncated deflections caused by steps (***a*, *d*, *e***) and ramp (***b*, *c*, *f***) commands. Inset shows an example image of a cell with recording pipette. (***B***) Step-elicited currents, taken at *a*, *d* indicated in trace *A*, exhibited a robust delayed outward rectification and a significant inward rectification, the latter was suppressed by 0.1 mM Ba^2+^. Each trace was averaged from two trials. (***C***) I/V curves constructed at times (***b*, *c***) in *A*, showing that Ba^2+^ suppressed the inward rectification (G_slope_ at -120 mV from 1.6 to 0.55 nS). (***D***) I/V plot of Ba^2+^-sensitive net current (subtractions of ***b—c***), showing that the current has a reversal potential (V_r_) near the calculated E_K_ (E_K_ = -85). The dashed curve resulted from fitting of the Boltzmann function revealing a maximal conductance G_max_ = 1.26 nS, a half activation voltage V_0.5_ = -66 mV, slope factor *k* = 12 mV/e-fold, and reversal potential V_r-Kir_ = -83 mV. Note that the Kir current has a voltage window between -83 and 0 mV, which allows outward K^+^ current flow.
(TIF)

S3 Fig Some VSMCs lack Ba^2+^-sensitive Kir conductance. (***A***) Holding current trace with large deflections caused by steps (***a*, *d*, *e*, *g*, *j*, *k***) and ramp (***b*, *c*, *f*, *h*, *i*, *l***) commands. (***B***) Step-elicited currents, taken at *a—k* indicated in trace ***A*.** The cell initially exhibited a delayed outward rectification but no inward rectification, the former was suppressed by 1 mM TEA and 4-AP (*B*,*a* vs. *B*,*d*). Each trace averaged from two trials. (***C***) I/V curves constructed by ramp commands at ***c*, *f*, *h*, *i*** in trace *A*, showing that elevations of [K^+^]_o_ to 20 and 60 mM and 100 mM Ba^2+^ had little effect on both inward and outward rectifications. (***D***) I/V plots of 60 mM [K^+^]_o_-induced and Ba^2+^-sensitive net current from subtractions of (***h*** *—* ***c*** and ***i*** *—* ***h***), showing that 60 mM [K^+^]_o_ caused v-shaped inward current between -40 and 40 mV and a 7.8 mV depolarization (see zero current voltage in ***C***) and add Ba^2+^ caused no effect in the I/V relation. This cell was from a SMA segment (indicated by an arrow in the micrograph inset in ***C***) with a C_input_ of 15 pF.
(TIF)

S4 FigMeasurement of K_DR_ current parameters by Boltzmann function fit in a VSMC.Step-induced whole-cell currents in the boxed inset showed typical strong outward rectification with initial delayed time course. The ramp command constructed whole-cell I/V plot was fitted with the modified Boltzmann function between -60 and 78 mV: $I=\left(V_{m}+86\right)*\left(G_{\text{max}}-G_{\text{max}}/\left(1+\text{exp}\left(\left(V_{m}-V_{0.5}\right)/k\right)\right)\right)+0.73*V_{m}+C$, where the -86 mV was the calculated E_K_, the 0.73 nS were leakage conductance resulted from linear fit between -60 and -40 mV, C denoted a mathematic persistent resting current; and the last two items were for non-K_DR_ currents subtraction. The curve fit revealed: G_max_ = 3.0 nS, V_0.5_ = 45 mV, *k* = 19 mV/e-fold. Recordings were from a dispersed VSMC of the SMA. Data statistics of all cells from the SMA, BA and MA (n = 5, 3, 2) are G_max_ = 4.1 ± 0.33 nS, V_0.5_ = 38 ± 8.4 mV, *k* = 12 ± 6.5 mV/e-fold.(TIF)

S5 Fig Measurement of net K_ir_ current in a VSMC *in situ* of a BA segment. TEA (1 mM), nitrendipine (1 μM), glipizide (3 μM) and 18βGA (30 μM) were present throughout to minimize other K^+^- and Ca^2+^-currents and to isolate the recorded cell from gap junction couplings [40,41]. (***A***) Step voltage commands (-140 to 40 mV in 20 mV increments, inset at ***A***.***a***) induced whole-cell current traces. ***A*.*a***: currents in 0 mM K^+^ were taken as the residual background currents with near zero K_ir_ [52]. After K_ir_ current was enhanced by 60 mM K^+^ (***A***.***b***), the net K_ir_ current (***A***.***c***) was obtained by subtracting the residual currents (***A***.***a***) from the enhanced currents (***A***.***b***). ***A*.*d***: Traces showed that the net K_ir_ current (***A***.***c***) was completely blocked by 100 μM Ba^2+^. (***B***) I/V plots of the data from ***a*** *and* ***b*** in ***A***. (***C***) I/V plot of the net Kir current (***b—a***). (***D***) Ramp voltage-constructed I-V curves show a Kir-current similar to ***C***, which was completely blocked by 100 μM Ba^2+^. Note that the inward shift of the high K^+^ at its calculated E_K_ (-20 mV) was nullified in ***C*** and ***D***. Boltzmann function fit to the Ba^2+^-sensitive current revealed a maximal conductance of 3.9 nS, half activation voltage at -14 mV and a slope of 5.6 mV/e-fold.
(TIF)
