# Supplementary material for: Diverse Kir Expression Contributes to Distinct Bimodal Distribution of Resting Potentials and Vasotone Responses of Arterioles
Source: PLoS One. 2015 May 4;10(5):e0125266. doi: 10.1371/journal.pone.0125266 (PMC4418701; doi:10.1371/journal.pone.0125266)
Supplement: S1 Dataset — (DOCX) [file pone.0125266.s001.docx]

**Computation program lines for figure 8**

**SMA simulation**

clear all

close all

Vhd = 40.; kd = 12;

Vhk = -75; kk = 13;

Ek = -86; EL = 0;

Vm(1) = -40;

Nr = 10000;

Gmaxk = 0.1 + 0.01*randn(1,Nr);

Gnv = 0.01 + 0.001*randn(1,Nr);

GL = 0.018 + 0.01*randn(1,Nr);

Gmaxd = 4.0 + 0.4*randn(1,Nr);

Nit = 200;

for j=1:Nr,

Gk(1) = Gmaxk(j)./(1+exp((Vm(1)-Vhk)/kk));

Gd(1) = Gmaxd(j) - Gmaxd(j)./(1+exp((Vm(1)-Vhd)/kd));

for i=2:Nit,

Vm(i) = -0+(Ek*(Gd(i-1)+Gk(i-1)+Gnv(j))+0*GL(j))/(Gd(i-1)+ Gk(i-1)+Gnv(j)+GL(j));

Gk(i) = Gmaxk(j)./(1+exp((Vm(i)-Vhk)/kk));

Gd(i) = Gmaxd(j) - Gmaxd(j)./(1+exp((Vm(i)-Vhd)/kd));

end

V(j) = Vm(Nit);

end

**BA simulation**

clear all

close all

Vhd = 40.; kd = 12;

Vhk = -75; kk = 13;

Ek = -86; EL = 0;

Vm(1) = -40;

Nr = 10000;

Gmaxk = 0.2 + 0.02*randn(1,Nr);

Gnv = 0.01 + 0.001*randn(1,Nr);

GL = 0.018 + 0.01*randn(1,Nr);

Gmaxd = 4.0 + 0.4*randn(1,Nr);

Nit = 200;

for j=1:Nr,

Gk(1) = Gmaxk(j)./(1+exp((Vm(1)-Vhk)/kk));

Gd(1) = Gmaxd(j) - Gmaxd(j)./(1+exp((Vm(1)-Vhd)/kd));

for i=2:Nit,

Vm(i) = -0+(Ek*(Gd(i-1)+Gk(i-1)+Gnv(j))+0*GL(j))/(Gd(i-1)+ Gk(i-1)+Gnv(j)+GL(j));

Gk(i) = Gmaxk(j)./(1+exp((Vm(i)-Vhk)/kk));

Gd(i) = Gmaxd(j) - Gmaxd(j)./(1+exp((Vm(i)-Vhd)/kd));

end

V(j) = Vm(Nit);

end

**MA simulation**

clear all

close all

Vhd = 20.; kd = 12;

Vhk = -80; kk = 15;

Ek = -86; EL = 0;

Vm(1) = -40;

Nr = 10000;

Gmaxk = 0.2 + 0.02*randn(1,Nr);

Gnv = 0.01 + 0.001*randn(1,Nr);

GL = 0.018 + 0.01*randn(1,Nr);

Gmaxd = 4.0 + 0.4*randn(1,Nr);

Nit = 200;

for j=1:Nr,

Gk(1) = Gmaxk(j)./(1+exp((Vm(1)-Vhk)/kk));

Gd(1) = Gmaxd(j) - Gmaxd(j)./(1+exp((Vm(1)-Vhd)/kd));

for i=2:Nit,

Vm(i) = -0+(Ek*(Gd(i-1)+Gk(i-1)+Gnv(j))+0*GL(j))/(Gd(i-1)+ Gk(i-1)+Gnv(j)+GL(j));

Gk(i) = Gmaxk(j)./(1+exp((Vm(i)-Vhk)/kk));

Gd(i) = Gmaxd(j) - Gmaxd(j)./(1+exp((Vm(i)-Vhd)/kd));

end

V(j) = Vm(Nit);

end

**K_NV_ Up**

clear all

close all

Vhd = 40.; kd =12;

Vhk = -75; kk = 13;

Ek = -86; EL = 0;

Vm(1) = -40;

Nr = 10000;

Gmaxk = 0.1 + 0.01*randn(1,Nr);

Gnv = 0.03 + 0.003*randn(1,Nr);

GL = 0.018 + 0.01*randn(1,Nr);

Gmaxd = 4.0 + 0.4*randn(1,Nr);

Nit = 200;

for j=1:Nr,

Gk(1) = Gmaxk(j)./(1+exp((Vm(1)-Vhk)/kk));

Gd(1) = Gmaxd(j) - Gmaxd(j)./(1+exp((Vm(1)-Vhd)/kd));

for i=2:Nit,

Vm(i) = -0+(Ek*(Gd(i-1)+Gk(i-1)+Gnv(j))+0*GL(j))/(Gd(i-1)+ Gk(i-1)+Gnv(j)+GL(j));

Gk(i) = Gmaxk(j)./(1+exp((Vm(i)-Vhk)/kk));

Gd(i) = Gmaxd(j) - Gmaxd(j)./(1+exp((Vm(i)-Vhd)/kd));

end

V(j) = Vm(Nit);

end

**I_L_ Up**

clear all

close all

Vhd = 40.; kd =12;

Vhk = -75; kk = 13;

Ek = -86; EL = 0;

Vm(1) = -40;

Nr = 10000;

Gmaxk = 0.1 + 0.01*randn(1,Nr);

Gnv = 0.01 + 0.001*randn(1,Nr);

GL = 0.02 + 0.01*randn(1,Nr);

Gmaxd = 4.0 + 0.4*randn(1,Nr);

Nit = 200;

for j=1:Nr,

Gk(1) = Gmaxk(j)./(1+exp((Vm(1)-Vhk)/kk));

Gd(1) = Gmaxd(j) - Gmaxd(j)./(1+exp((Vm(1)-Vhd)/kd));

for i=2:Nit,

Vm(i) = -0+(Ek*(Gd(i-1)+Gk(i-1)+Gnv(j))+0*GL(j))/(Gd(i-1)+ Gk(i-1)+Gnv(j)+GL(j));

Gk(i) = Gmaxk(j)./(1+exp((Vm(i)-Vhk)/kk));

Gd(i) = Gmaxd(j) - Gmaxd(j)./(1+exp((Vm(i)-Vhd)/kd));

end

V(j) = Vm(Nit);

end

**K_D_ Up**

clear all

close all

Vhd = 40.; kd =12;

Vhk = -75; kk = 13;

Ek = -86; EL = 0;

Vm(1) = -40;

Nr = 10000;

Gmaxk = 0.1 + 0.01*randn(1,Nr);

Gnv = 0.01 + 0.001*randn(1,Nr);

GL = 0.018 + 0.01*randn(1,Nr);

Gmaxd = 8.0 + 0.8*randn(1,Nr);

Nit = 200;

for j=1:Nr,

Gk(1) = Gmaxk(j)./(1+exp((Vm(1)-Vhk)/kk));

Gd(1) = Gmaxd(j) - Gmaxd(j)./(1+exp((Vm(1)-Vhd)/kd));

for i=2:Nit,

Vm(i) = -0+(Ek*(Gd(i-1)+Gk(i-1)+Gnv(j))+0*GL(j))/(Gd(i-1)+ Gk(i-1)+Gnv(j)+GL(j));

Gk(i) = Gmaxk(j)./(1+exp((Vm(i)-Vhk)/kk));

Gd(i) = Gmaxd(j) - Gmaxd(j)./(1+exp((Vm(i)-Vhd)/kd));

end

V(j) = Vm(Nit);

end

**K_NV_ Down**

clear all

close all

Vhd = 40.; kd =12;

Vhk = -75; kk = 13;

Ek = -86; EL = 0;

Vm(1) = -40;

Nr = 10000;

Gmaxk = 0.1 + 0.01*randn(1,Nr);

Gnv = 0.005 + 0.0005*randn(1,Nr);

GL = 0.018 + 0.01*randn(1,Nr);

Gmaxd = 4.0 + 0.4*randn(1,Nr);

Nit = 200;

for j=1:Nr,

Gk(1) = Gmaxk(j)./(1+exp((Vm(1)-Vhk)/kk));

Gd(1) = Gmaxd(j) - Gmaxd(j)./(1+exp((Vm(1)-Vhd)/kd));

for i=2:Nit,

Vm(i) = -0+(Ek*(Gd(i-1)+Gk(i-1)+Gnv(j))+0*GL(j))/(Gd(i-1)+ Gk(i-1)+Gnv(j)+GL(j));

Gk(i) = Gmaxk(j)./(1+exp((Vm(i)-Vhk)/kk));

Gd(i) = Gmaxd(j) - Gmaxd(j)./(1+exp((Vm(i)-Vhd)/kd));

end

V(j) = Vm(Nit);

end

**I_L_ Down**

clear all

close all

Vhd = 40.; kd =12;

Vhk = -75; kk = 13;

Ek = -86; EL = 0;

Vm(1) = -40;

Nr = 10000;

Gmaxk = 0.1 + 0.01*randn(1,Nr);

Gnv = 0.01 + 0.001*randn(1,Nr);

GL = 0.010 + 0.005*randn(1,Nr);

Gmaxd = 4.0 + 0.4*randn(1,Nr);

Nit = 200;

for j=1:Nr,

Gk(1) = Gmaxk(j)./(1+exp((Vm(1)-Vhk)/kk));

Gd(1) = Gmaxd(j) - Gmaxd(j)./(1+exp((Vm(1)-Vhd)/kd));

for i=2:Nit,

Vm(i) = -0+(Ek*(Gd(i-1)+Gk(i-1)+Gnv(j))+0*GL(j))/(Gd(i-1)+ Gk(i-1)+Gnv(j)+GL(j));

Gk(i) = Gmaxk(j)./(1+exp((Vm(i)-Vhk)/kk));

Gd(i) = Gmaxd(j) - Gmaxd(j)./(1+exp((Vm(i)-Vhd)/kd));

end

V(j) = Vm(Nit);

end

**Kir Down**

clear all

close all

Vhd = 40.; kd =12;

Vhk = -75; kk = 13;

Ek = -86; EL = 0;

Vm(1) = -40;

Nr = 10000;

Gmaxk = 0.05 + 0.005*randn(1,Nr);

Gnv = 0.01 + 0.001*randn(1,Nr);

GL = 0.018 + 0.01*randn(1,Nr);

Gmaxd = 4.0 + 0.4*randn(1,Nr);

Nit = 200;

for j=1:Nr,

Gk(1) = Gmaxk(j)./(1+exp((Vm(1)-Vhk)/kk));

Gd(1) = Gmaxd(j) - Gmaxd(j)./(1+exp((Vm(1)-Vhd)/kd));

for i=2:Nit,

Vm(i) = -0+(Ek*(Gd(i-1)+Gk(i-1)+Gnv(j))+0*GL(j))/(Gd(i-1)+ Gk(i-1)+Gnv(j)+GL(j));

Gk(i) = Gmaxk(j)./(1+exp((Vm(i)-Vhk)/kk));

Gd(i) = Gmaxd(j) - Gmaxd(j)./(1+exp((Vm(i)-Vhd)/kd));

end

V(j) = Vm(Nit);

end
