# Supplementary material for: Diverse Kir Expression Contributes to Distinct Bimodal Distribution of Resting Potentials and Vasotone Responses of Arterioles
Source: PLoS One. 2015 May 4;10(5):e0125266. doi: 10.1371/journal.pone.0125266 (PMC4418701; doi:10.1371/journal.pone.0125266)
Supplement: S2 Dataset — (DOC) [file pone.0125266.s002.doc]

**Computation program lines for Figure 9**

**Kir inhibition**

clear all

% close all

Vhd = 40; kd =12;

Vhk = -70; kk =8; %9

Ek = -86; Ep =-160; EL=0;

Nr = 200;

Nc = 30;

t1 = [1:Nc*Nr]/Nr;

%Gmaxk = 0.2;

Gbegin = 0.2; %0.25

Gmin = 0.1; %0.15

Gend = 0.2; %0.25

Gmaxk(1:Nc*Nr) = (Gbegin-Gmin)./(1+exp((t1-10)/1.5))+Gmin;

Gmaxk(Nc*Nr+1:Nc*Nr*2) = (Gend-Gmin)./(1+exp((10-t1)/1.5))+Gmin;

t=[t1 t1+Nc];

plot(t,Gmaxk)

GL = 0.02;

Gnv = 0.01;

Gmaxd = 4.0 ;

Nit = 800;

Nf = 0;

Vm(1) = -70;

for j=1:Nc*Nr*2,

Gk(1) = Gmaxk(j)/(1+exp((Vm(1)-Vhk)/kk));

Gd(1) = Gmaxd - Gmaxd./(1+exp((Vm(1)-Vhd)/kd));

for i=2:Nit,

Vm(i) = (Ek*(Gd(i-1)+Gk(i-1)+Gnv)+0*GL)/(Gd(i-1)+Gk(i-1)+GL+Gnv);

Gk(i) = Gmaxk(j)./(1+exp((Vm(i)-Vhk)/kk));

Gd(i) = Gmaxd - Gmaxd./(1+exp((Vm(i)-Vhd)/kd));

if abs(Vm(i)-Vm(i-1)) < 0.01*abs(Vm)

break;

end

end

if abs(Vm(i)-Vm(i-1)) < 0.01*abs(Vm),

V(j) = Vm(i);

Vm(1) = V(j);

else

Nf = Nf +1;

end

end

**K_NV_ activation**

clear all

%close all

Vhd = 40.; kd =12;

Vhk = -70; kk = 12;

Ek = -86; EL = 0;

Nr = 200;

Nc = 30;

t1 = [1:Nc*Nr]/Nr;

Gbegin = 0.01;

Gmax = 0.016;

Gend = 0.01;

Gnv(1:Nc*Nr) = (Gmax-Gbegin)./(1+exp(10-t1))+Gbegin;

Gnv(Nc*Nr+1:Nc*Nr*2) = (Gmax-Gend)./(1+exp(t1-10))+Gend;

t=[t1 t1+Nc];

plot(t,Gnv)

GL = 0.02;

Gmaxk = 0.05;

Gmaxd = 4 ;

Nit = 800;

Nf = 0;

Vm(1) = -30;

for j=1:Nc*Nr*2,

Gk(1) = Gmaxk/(1+exp(Vm(1)-Vhk)/kk);

Gd(1) = Gmaxd - Gmaxd./(1+exp(Vm(1)-Vhd)/kd);

for i=2:Nit,

Vm(i) = (Ek*(Gd(i-1)+Gk(i-1)+Gnv(j))+0*GL)/(Gd(i-1)+Gk(i-1)+ GL);

Gk(i) = Gmaxk./(1+exp(Vm(i)-Vhk)/kk);

Gd(i) = Gmaxd - Gmaxd./(1+exp(Vm(i)-Vhd)/kd);

if abs(Vm(i)-Vm(i-1)) < 0.01*abs(Vm)

break;

end

end

if abs(Vm(i)-Vm(i-1)) < 0.01*abs(Vm),

V(j) = Vm(i);

Vm(1) = V(j);

else

Nf = Nf +1;

end

end
