# Supplementary figures and images for: Diverse Kir Expression Contributes to Distinct Bimodal Distribution of Resting Potentials and Vasotone Responses of Arterioles
Source: PLoS One. 2015 May 4;10(5):e0125266. doi: 10.1371/journal.pone.0125266 (PMC4418701; doi:10.1371/journal.pone.0125266)

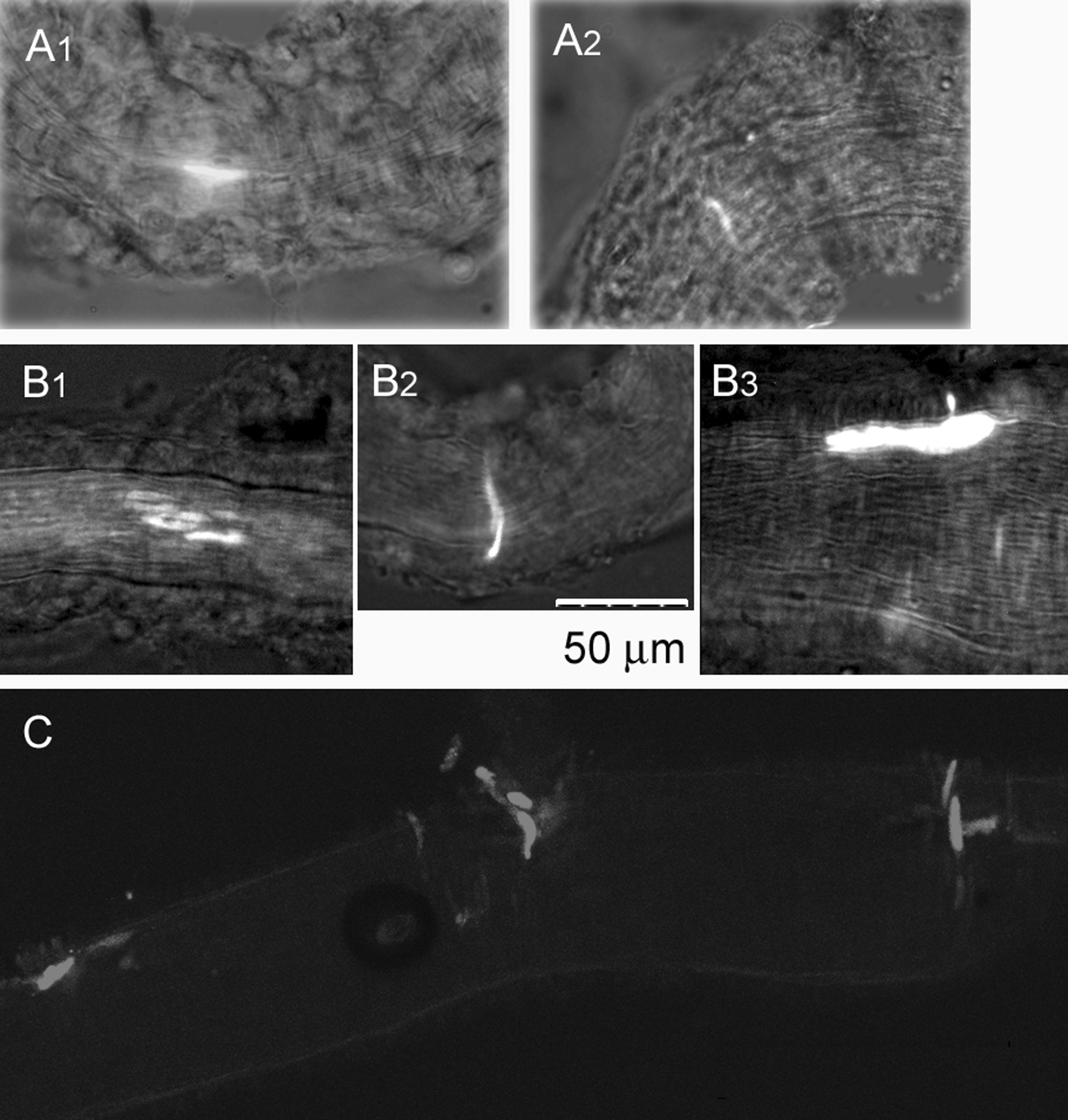

Supplement: S1 Fig — Propidium iodide (PI) was used in electrode filling solution to label nucleus of the arteriolar cells. (A and B) Micrographs were taken with simultaneous fluorescent and DIC illumination to show the labeled cells and vessel contours of the SMA (A) and MA (B). A1 & B1 depict the intercellular dye-transfer between ECs, B2 between VSMCs, and B3 showing between EC and SMC. (C) is reconstruction of confocal images from a BA (AICA branch) showing EC-EC, SMC-SMC and SMC-EC dye-couplings. VSMC-VSMC (2/68) and VSMC-EC (1/68) dye-coupling were less frequently detected in the SMA than the other two vessel types. (TIF) [file pone.0125266.s003.tif]

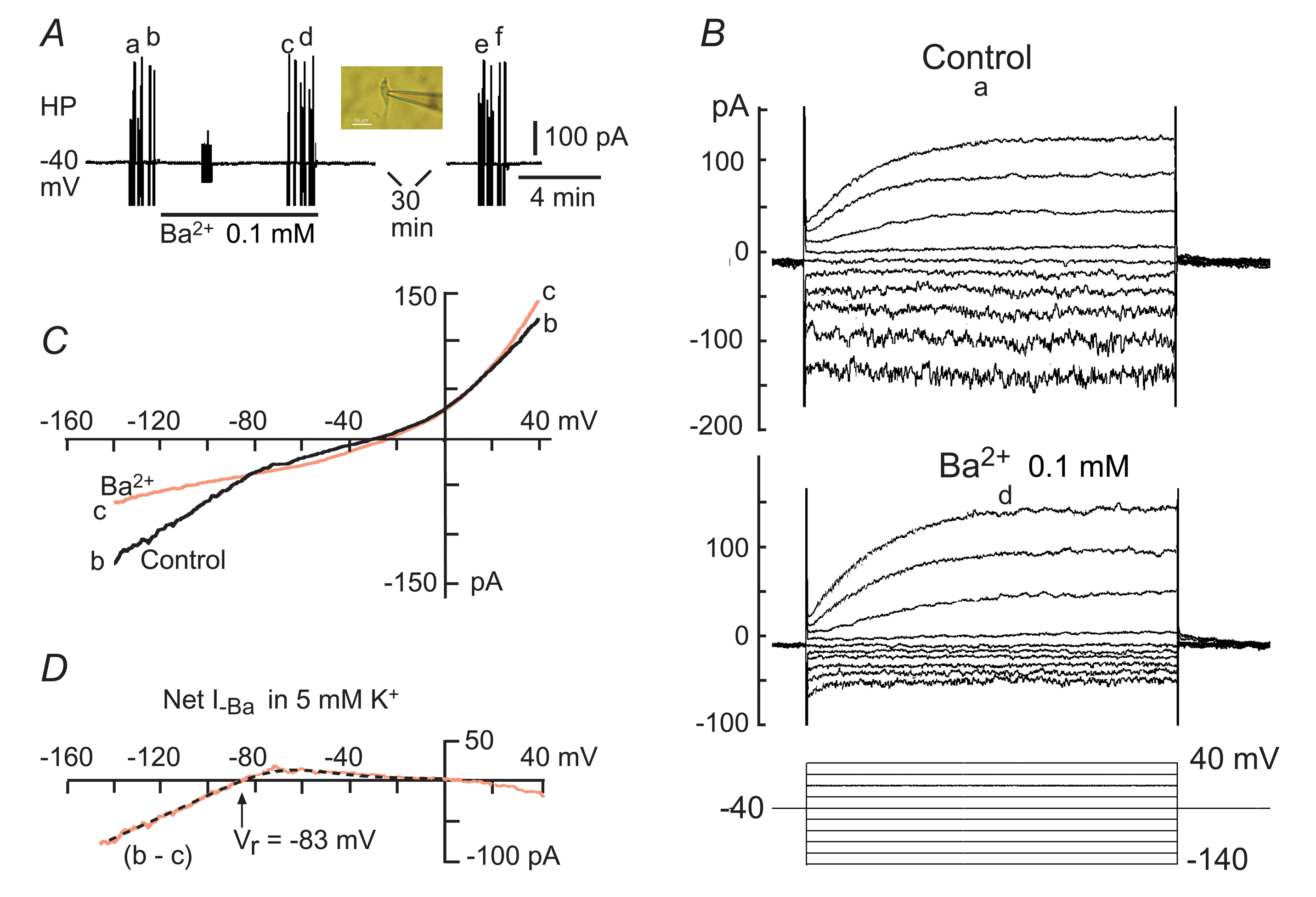

Supplement: S2 Fig — (A) Holding current trace with truncated deflections caused by steps (a, d, e) and ramp (b, c, f) commands. Inset shows an example image of a cell with recording pipette. (B) Step-elicited currents, taken at a, d indicated in trace A, exhibited a robust delayed outward rectification and a significant inward rectification, the latter was suppressed by 0.1 mM Ba2+. Each trace was averaged from two trials. (C) I/V curves constructed at times (b, c) in A, showing that Ba2+ suppressed the inward rectification (Gslope at -120 mV from 1.6 to 0.55 nS). (D) I/V plot of Ba2+-sensitive net current (subtractions of b—c), showing that the current has a reversal potential (Vr) near the calculated EK (EK = -85). The dashed curve resulted from fitting of the Boltzmann function revealing a maximal conductance Gmax = 1.26 nS, a half activation voltage V0.5 = -66 mV, slope factor k = 12 mV/e-fold, and reversal potential Vr-Kir = -83 mV. Note that the Kir current has a voltage window between -83 and 0 mV, which allows outward K+ current flow. (TIF) [file pone.0125266.s004.tif]

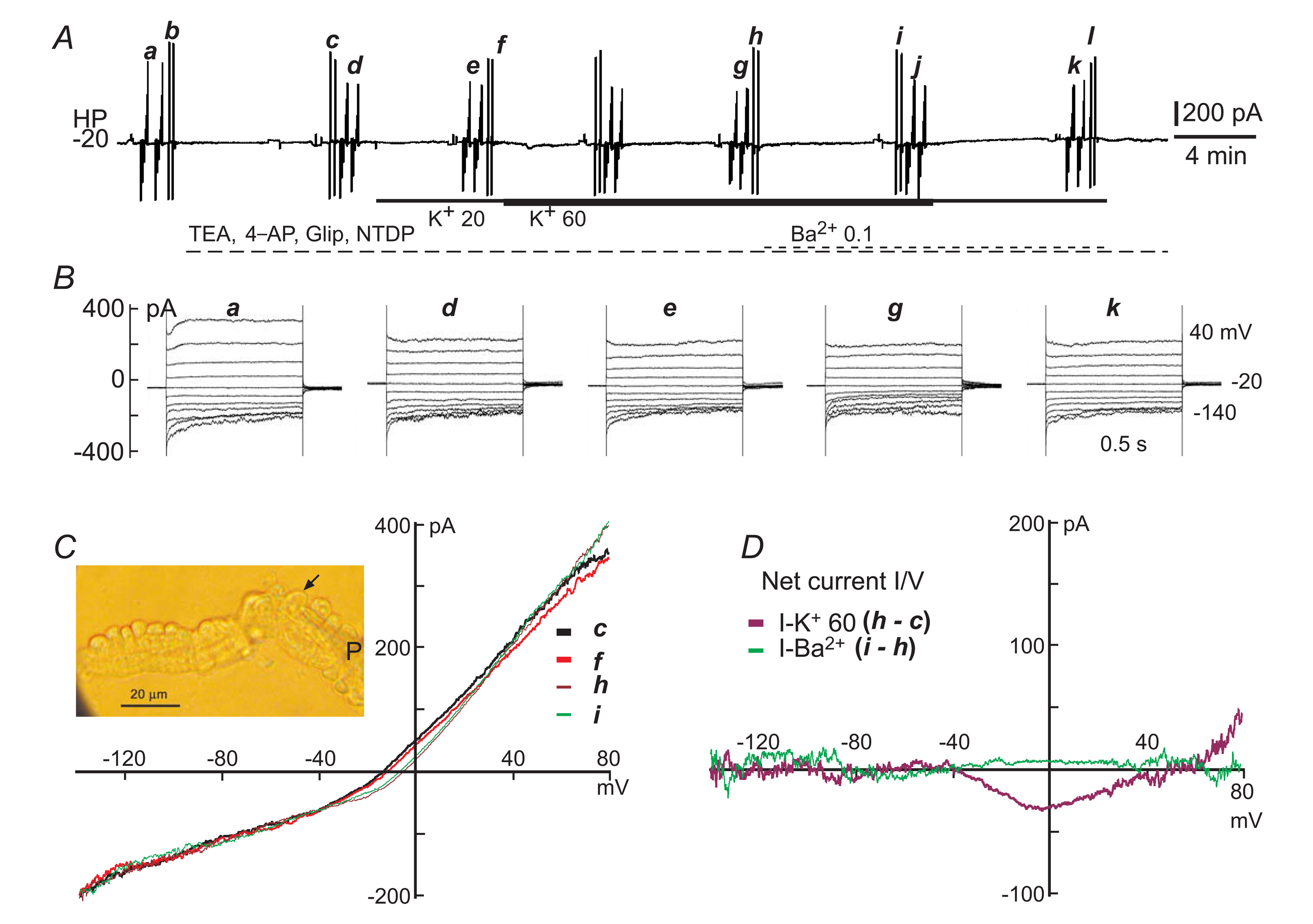

Supplement: S3 Fig — (A) Holding current trace with large deflections caused by steps (a, d, e, g, j, k) and ramp (b, c, f, h, i, l) commands. (B) Step-elicited currents, taken at a—k indicated in trace A. The cell initially exhibited a delayed outward rectification but no inward rectification, the former was suppressed by 1 mM TEA and 4-AP (B,a vs. B,d). Each trace averaged from two trials. (C) I/V curves constructed by ramp commands at c, f, h, i in trace A, showing that elevations of [K+]o to 20 and 60 mM and 100 mM Ba2+ had little effect on both inward and outward rectifications. (D) I/V plots of 60 mM [K+]o-induced and Ba2+-sensitive net current from subtractions of (h — c and i — h), showing that 60 mM [K+]o caused v-shaped inward current between -40 and 40 mV and a 7.8 mV depolarization (see zero current voltage in C) and add Ba2+ caused no effect in the I/V relation. This cell was from a SMA segment (indicated by an arrow in the micrograph inset in C) with a Cinput of 15 pF. (TIF) [file pone.0125266.s005.tif]

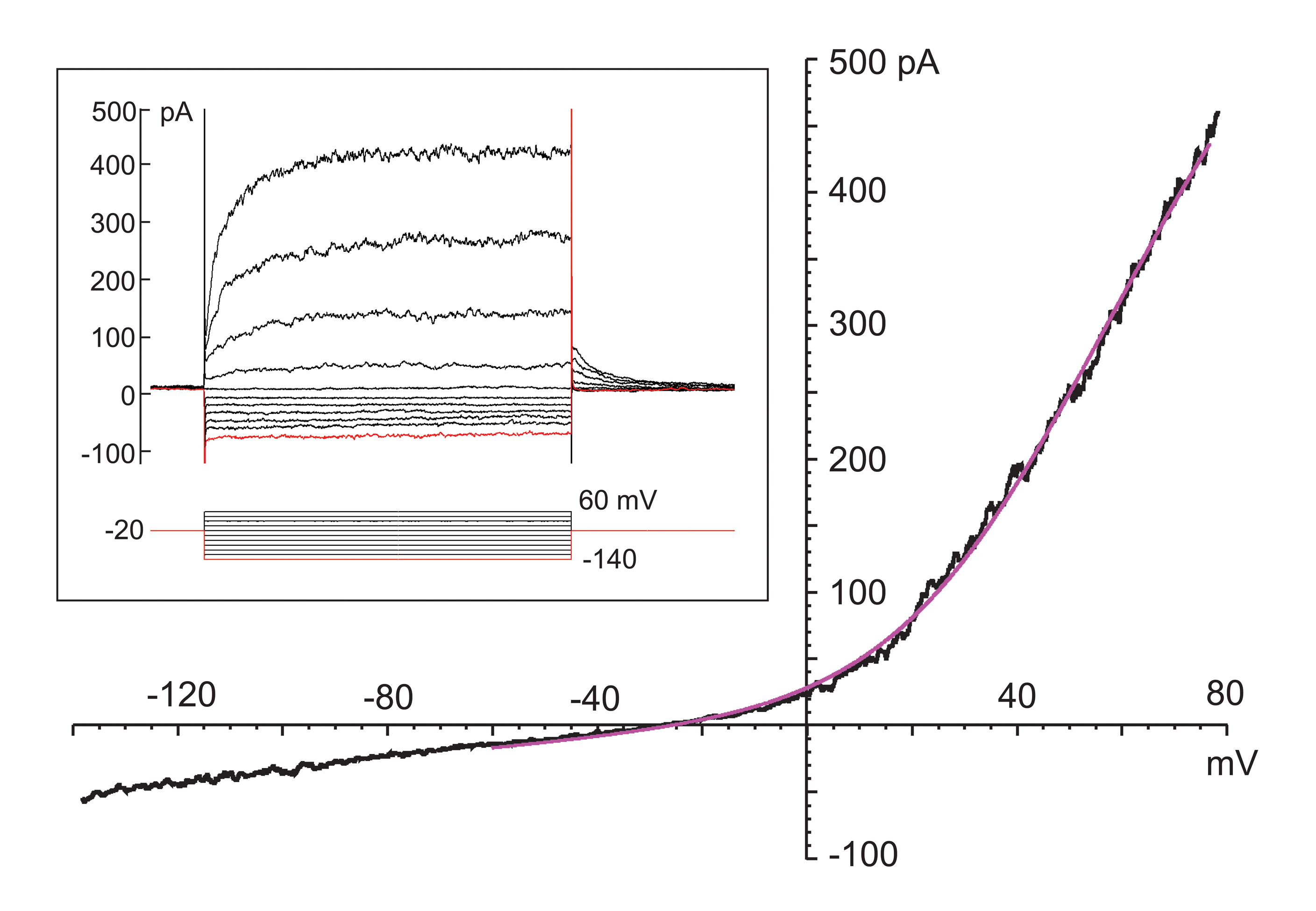

Supplement: S4 Fig — Step-induced whole-cell currents in the boxed inset showed typical strong outward rectification with initial delayed time course. The ramp command constructed whole-cell I/V plot was fitted with the modified Boltzmann function between -60 and 78 mV: I=(Vm+86)*(Gmax−Gmax/(1+exp((Vm−V0.5)/k)))+0.73*Vm+C, where the -86 mV was the calculated EK, the 0.73 nS were leakage conductance resulted from linear fit between -60 and -40 mV, C denoted a mathematic persistent resting current; and the last two items were for non-KDR currents subtraction. The curve fit revealed: Gmax = 3.0 nS, V0.5 = 45 mV, k = 19 mV/e-fold. Recordings were from a dispersed VSMC of the SMA. Data statistics of all cells from the SMA, BA and MA (n = 5, 3, 2) are Gmax = 4.1 ± 0.33 nS, V0.5 = 38 ± 8.4 mV, k = 12 ± 6.5 mV/e-fold. (TIF) [file pone.0125266.s006.tif]

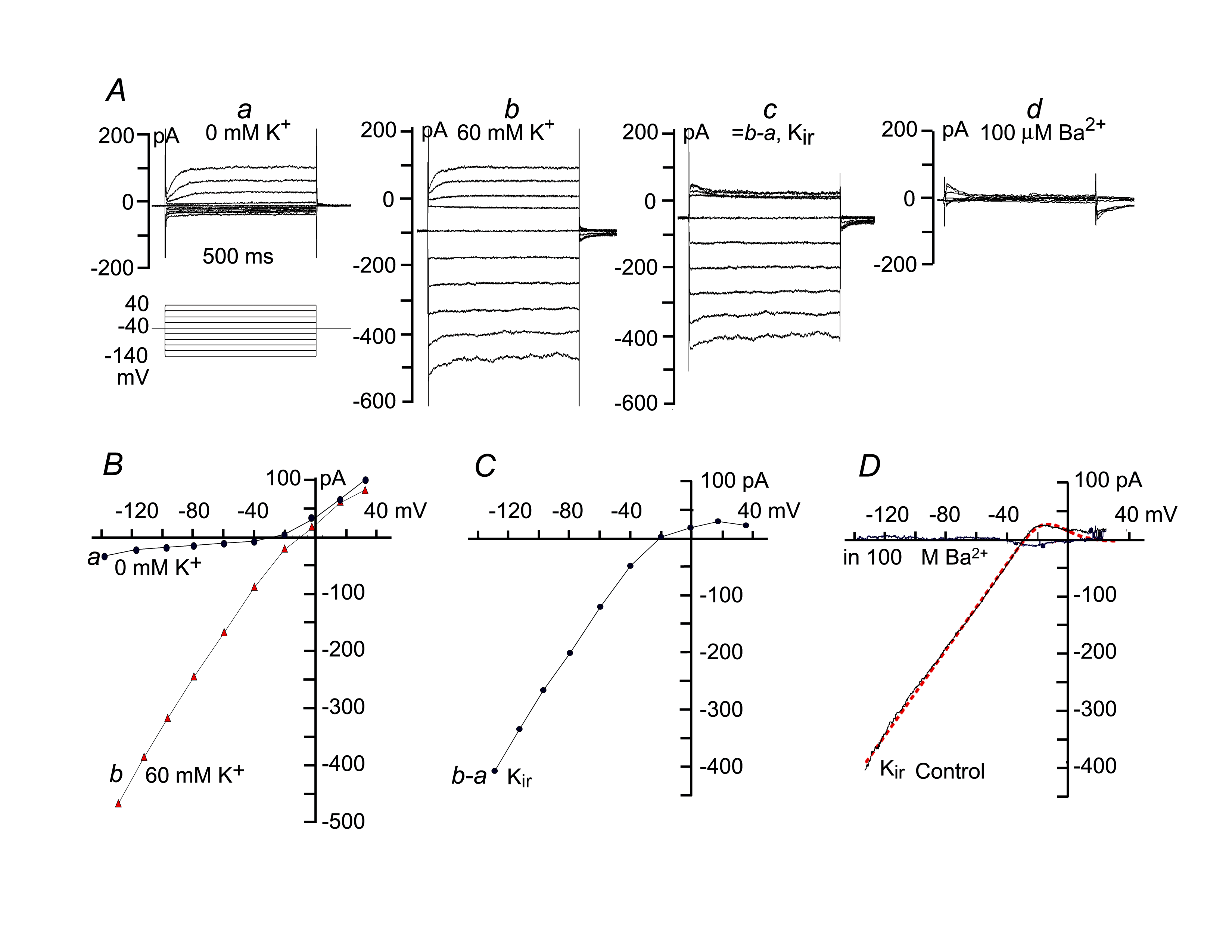

Supplement: S5 Fig — TEA (1 mM), nitrendipine (1 μM), glipizide (3 μM) and 18βGA (30 μM) were present throughout to minimize other K+- and Ca2+-currents and to isolate the recorded cell from gap junction couplings [40,41]. (A) Step voltage commands (-140 to 40 mV in 20 mV increments, inset at A.a) induced whole-cell current traces. A.a: currents in 0 mM K+ were taken as the residual background currents with near zero Kir [52]. After Kir current was enhanced by 60 mM K+ (A.b), the net Kir current (A.c) was obtained by subtracting the residual currents (A.a) from the enhanced currents (A.b). A.d: Traces showed that the net Kir current (A.c) was completely blocked by 100 μM Ba2+. (B) I/V plots of the data from a and b in A. (C) I/V plot of the net Kir current (b—a). (D) Ramp voltage-constructed I-V curves show a Kir-current similar to C, which was completely blocked by 100 μM Ba2+. Note that the inward shift of the high K+ at its calculated EK (-20 mV) was nullified in C and D. Boltzmann function fit to the Ba2+-sensitive current revealed a maximal conductance of 3.9 nS, half activation voltage at -14 mV and a slope of 5.6 mV/e-fold. (TIF) [file pone.0125266.s007.tif]
